# Chemical Design of Functional Polymer Structures for Biosensors: From Nanoscale to Macroscale

**DOI:** 10.3390/polym10050551

**Published:** 2018-05-21

**Authors:** Kyoung Min Lee, Kyung Ho Kim, Hyeonseok Yoon, Hyungwoo Kim

**Affiliations:** 1 Department of Polymer Engineering, Graduate School, Chonnam National University, 77 Yongbong-ro, Buk-gu, Gwangju 61186, Korea; dlrudals999@snu.ac.kr (K.M.L.); doublekh0119@gmail.com (K.H.K.); 2 Department of Materials Science and Engineering, Seoul National University, 1 Gwanak-ro, Gwanak-gu, Seoul 08826, Korea; 3 School of Polymer Science and Engineering, Chonnam National University, 77 Yongbong-ro, Buk-gu, Gwangju 61186, Korea

**Keywords:** functional polymers, nanoparticles, hydrogels, chemical structures, biosensor

## Abstract

Over the past decades, biosensors, a class of physicochemical detectors sensitive to biological analytes, have drawn increasing interest, particularly in light of growing concerns about human health. Functional polymeric materials have been widely researched for sensing applications because of their structural versatility and significant progress that has been made concerning their chemistry, as well as in the field of nanotechnology. Polymeric nanoparticles are conventionally used in sensing applications due to large surface area, which allows rapid and sensitive detection. On the macroscale, hydrogels are crucial materials for biosensing applications, being used in many wearable or implantable devices as a biocompatible platform. The performance of both hydrogels and nanoparticles, including sensitivity, response time, or reversibility, can be significantly altered and optimized by changing their chemical structures; this has encouraged us to overview and classify chemical design strategies. Here, we have organized this review into two main sections concerning the use of nanoparticles and hydrogels (as polymeric structures) for biosensors and described chemical approaches in relevant subcategories, which act as a guide for general synthetic strategies.

## 1. Introduction

Polymers have advantages over metallic and ceramic materials for biosensing applications, such as mild synthetic conditions, scalable and large-area processing, chemical and structural diversity, flexibility, low operating temperature, and biocompatibility. Progress in nanotechnology has provided opportunities to design and fabricate various polymer nanostructures for biosensing applications. Several key requirements of polymeric materials are necessary to expand their applications. The most important parameters for sensing performance are sensitivity, response/recovery time, and reversibility/reproducibility of response, which are strongly dependent on the chemical structure and size of the polymers [1,2]. In addition, selectivity toward a specific target and the prevention of non-specific binding or adsorption are prerequisites for precise detection.

Nanoparticles are of great importance in sensing applications because of their large, effective surface area, which increases the rate of response, as well as the sensitivity. In the past few decades, rapid progress in nanotechnology has occurred, and this has stimulated the development of nanoparticles. Thus, we can now not only optimize the chemical or physical properties of the particles on demand but also manipulate the nanostructure or morphology, achieving an optimized sensing system. For in vivo biosensing applications, the toxicity of nanoparticles is a critical factor to consider. Nanoparticles can be engineered with various surface functional groups and bioconjugates to enhance their biocompatibility [3].

On the other hand, stimuli-responsive polymeric materials have been investigated on a large scale for practical use [4]. In particular, interest in hydrogels has increased, and they have been frequently used in healthcare devices [5]. Hydrogels confine a considerable amount of water, which provides biocompatibility as well as biodegradability. In addition, the hydrogel-based, macroscale materials are known to be elastic yet durable, and transparent, all necessary properties of prosthetic devices. Furthermore, these materials are functionally alterable, allowing the preparation of optimized and bespoke materials for application in wearable or even implantable devices [6].

In this review paper, we focus on the design principles and chemical strategies for the preparation of polymeric materials from the perspective of synthetic chemistry and materials science using recent, notable examples. These materials have been designed to achieve the desired properties in the biosensing applications and would lead to a prosthetic polymer-based nervous system in the future (e.g., artificial human skin).

## 2. Nanostructure: Nanoparticles

**Figure 1:**
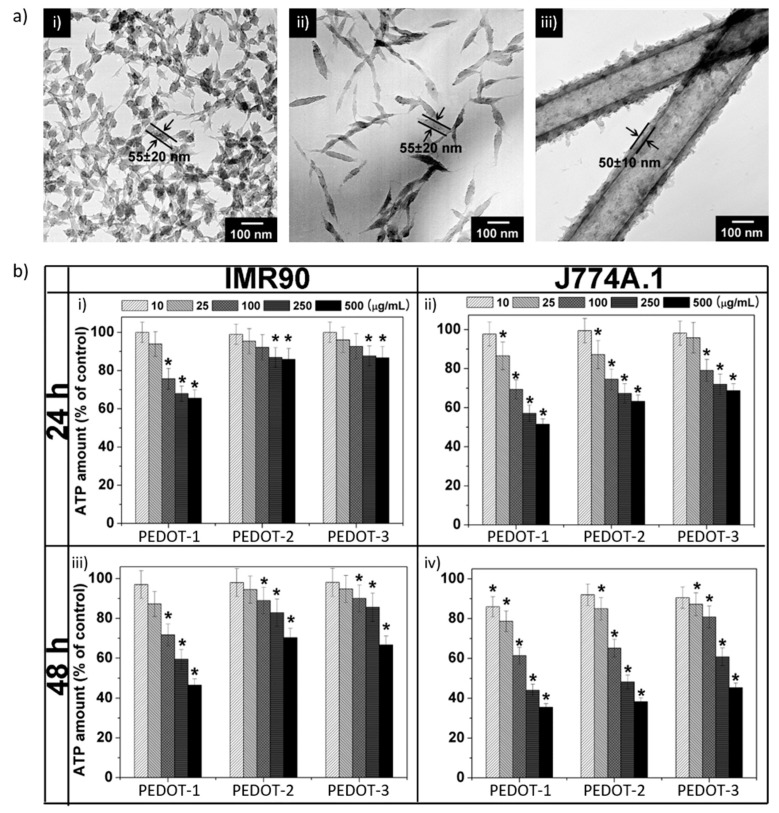
(**a**) Transmission electron microscope (TEM) images of poly(3,4-ethylenedioxythiophene) (PEDOT) nanomaterials of three different shapes: (**i**) PEDOT-1; (**ii**) PEDOT-2; and (**iii**) PEDOT-3; (**b**) Viability of fibroblast (IMR90) and macrophage (J774A.1) cells in the presence of PEDOT nanomaterials, which was determined by the amount of ATP in the cells. IMR90 cells were incubated with PEDOT nanomaterials for (**i**) 24 and (**iii**) 48 h; J774A.1 cells for (**ii**) 24 and (**iv**) 48 h. Values expressed as the mean ± standard deviation (SD), and each experiment was performed in triplicate. * Statistically significant difference from control exposed to PEDOT nanomaterials (*p* < 0.05). Reproduced with permission from [12]. Copyright 2010, John Wiley & Sons.

With the rapid progress in nanotechnology, a variety of nanostructures have been developed for sensing applications. Compared to their bulk counterparts, nanoparticles have enhanced the effective surface area and small dimensions, which result in increased sensitivity and faster response times for the nanoparticle-based sensors. Control over the morphology and microstructures of the nanoparticles in biosensor applications is also crucial [7,8]. It is well known that the major chemical/physical properties of the nanoparticles depend on the morphology and microstructures [9,10,11]. Furthermore, the biocompatibility of the nanoparticles is also affected by the morphological characteristics. As a typical example, Jang and co-workers reported that poly(3,4-ethylenedioxythiophene) (PEDOT) nanostructures of different shapes (aspect ratios) exhibited aspect-ratio dependent cell viability and cytotoxicity (Figure 1) [12]. Briefly, the cytotoxicity and apoptosis of the PEDOT nanoparticles increased with their decreasing aspect ratio for both cell lines, and the formation of reactive oxygen species in cells treated with the PEDOT nanoparticles also depended on the shape and concentration of the nanoparticles. Therefore, for nanoparticle biosensing applications, the parameters affecting biocompatibility must be considered, as well as developing a level of biofunctionality to interact passively or actively with the surrounding biological species/processes.

Compared to inorganic semiconductors and metals, polymers are structurally unstable at the nanoscale, which makes it difficult to prepare polymer nanoparticles with controlled sizes and morphologies. Nevertheless, diverse methods have been developed to produce polymer nanostructures, such as the so-called template and template-free approaches. The templates encompass porous membranes, micelles, vesicles, and macromolecules. For example, Feng group has successfully fabricated mesoporous conducting polymer nanosheets using block copolymer templates [13,14,15]. On the other hand, template-free approaches utilize the self-assembly of molecules or nanoscale building blocks in the absence of any templates. These methods for fabricating polymer nanostructures have been well summarized and described in several review articles [16,17]. Importantly, most polymer nanoparticles require additional synthetic procedures when their inherent chemical structure is not appropriate for biosensor applications. The preparation of polymers with functionalities that yield antifouling behavior and selectivity in biosensing applications can be accomplished through physical and chemical routes, such as the incorporation of dopants into polymers, direct polymerization of monomers with functional groups, and post-polymerization modification. The following subsection addresses the strategies that have been used to design and prepare polymer nanostructures for biosensing applications.

### 2.1. Physical Doping

Functional components can be doped into polymers, allowing further chemical functionalization of the polymers under a controlled environment. In particular, conducting polymers have electronic/electrical properties that depend on the nature and incorporated status of the dopant ions [8]. Importantly, appropriate chemical functionalities can be incorporated into the polymers via the doping process. Dopants can be trapped in the polymer, and the doping/dedoping process is reversibly controlled. It should also be noted that the nature of the dopant can affect the biocompatibility of conducting polymers. Wallace and co-workers reported the influence of biological compounds as the dopant on the biocompatibility and biofunctionality of PEDOT. They found that dopants such as dextran sulfate, chondroitin sulfate, and alginate significantly affected the biologically relevant properties of the polymer (e.g., nanoroughness, modulus, wettability, and electrical impedance) and *in turn* biological interactions (e.g., cell adhesion) [18].

**Figure 2:**
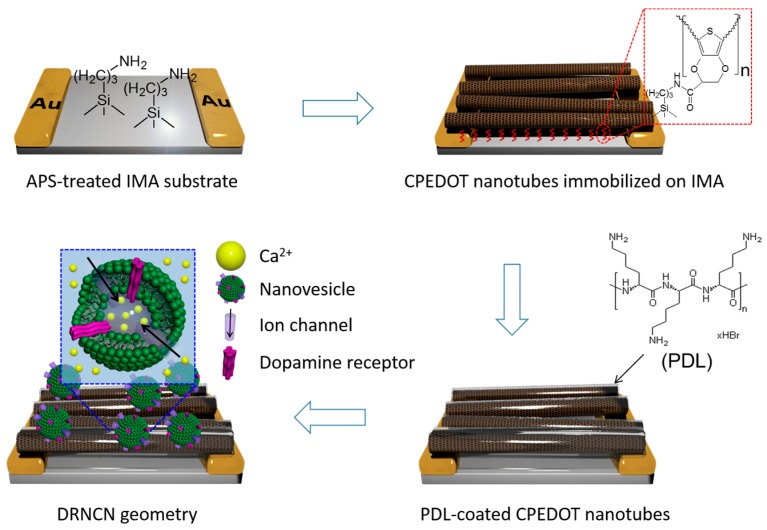
Schematic illustration of construction steps for dopamine-receptor-containing nanovesicle-immobilized carboxylated PEDOT (CPEDOT) nanotubes (DRNCNs) geometry. Reproduced with permission from [19]. Copyright 2014, Nature Publishing Group.

**Figure 3:**
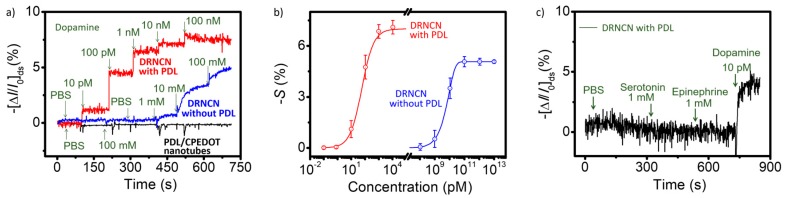
(**a**) Real-time responses with normalized current changes (Δ*I*/*I*_0_) and (**b**) calibration curves of DRNCN toward various dopamine concentrations (*S* indicates the normalized current change). (**c**) Selective responses of the dopamine biosensor using DRNCN toward non-target neurotransmitters (PBS, 1 mM Serotonin, and 1 mM Epinephrine) and dopamine (10 pM dopamine). Reproduced with permission [19]. Copyright 2014, Nature Publishing Group.

A good example of physical doping in biosensing has been demonstrated by Park and Jang [19]. A dopamine field-effect transistor (FET) biosensor was fabricated using dopamine-receptor-containing nanovesicle-immobilized carboxylated PEDOT (CPEDOT) nanotubes (DRNCNs) as a channel, where the nanovesicles acted as a gate-potential modulator. Figure 2 shows the fabrication process for the DRNCN FET sensor substrate. First, the CPEDOT nanotubes were attached to an interdigitated microelectrode array (IMA) via covalent coupling with the surface amino-silane group of the IMA and then, importantly, the nanovesicles were immobilized on the CPEDOT nanotubes with the aid of poly-d-lysine (PDL), which is a synthetic amino acid widely used as a coating to improve cell attachment because of its positive charge [20,21]. The resultant liquid-ion gated DRNCN FET sensor showed high sensitivity (minimum detectable level of as low as 10 pM) and high selectivity, even in human serum, as seen in Figure 3.

There are many candidate dopants that can affect the biological interactions of conducting polymers, such as glycosaminoglycans (e.g., chondroitin sulfate, heparin/heparin sulfate, and dextran sulfate) [22,23,24,25,26,27]. Those macromolecules all are negatively charged and, thus, can effectively serve as the dopant in conducting polymers. In addition, most polymers are amenable to physical doping, and a number of functional dopants are available, including peptides and proteins. However, unfortunately, undesirable dedoping can occur during sensing, which limits the applications of physical doping in biosensing.

### 2.2. Chemical Structure Modification of Monomers 

The most straightforward route to functional polymers for specific applications involves direct polymerization of monomers with the desired chemical structures and properties. Many commercial monomers are available from chemical manufacturers, derivatives of which can be synthesized via appropriate chemical reactions. From both quantitative and qualitative points of view, the direct use of monomers with more than a functionality (f-monomers) allows easy control on the incorporation of chemical functionalities into homopolymer chains. However, the important properties of the resulting polymers depend on the chemical structures, such as polarity, intra- or interchain interactions, and reactivity. Thus, additional effort should be devoted to the polymerization of f-monomers to control the size and morphology of the resulting nanoparticles. In some cases, f-monomers may prove incompatible with the polymerization conditions required to achieve high-molecular-weight polymers and to form well-defined nanoparticles with controlled sizes and morphologies. Furthermore, the reaction yield of the f-monomer may be too low to produce an appreciable quantity of the polymer for use, which reduces cost-effectiveness and efficiency. As a result, this approach significantly limits the flexibility in nanostructure design enabled by the broad range of polymerization techniques.

**Figure 4:**
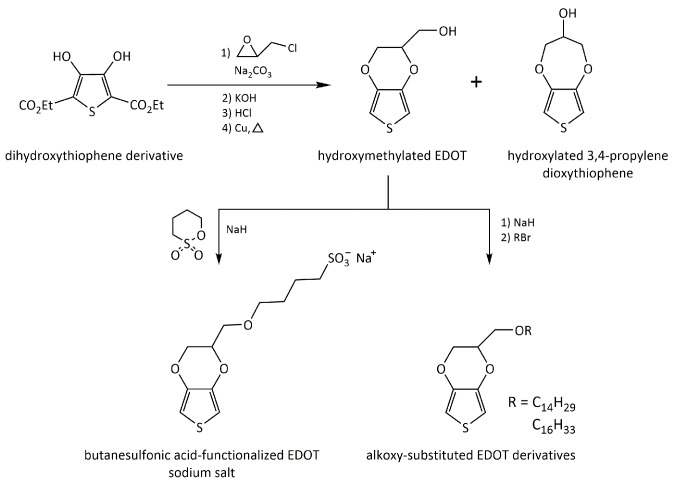
Synthetic schemes of several 3,4-ethylenedioxythiophene (EDOT) derivatives such as hydroxymethylated EDOT, sulfonatoalkoxy-substituted EDOT, and alkoxy-substituted EDOT [28].

Thiophene may be referred as the most prominent unit of derivatization to prepare f-monomers. There are many thiophene derivatives, including 3,4-ethylenedioxythiophene (EDOT) [28]. Polythiophene has attractive electrical and optical properties as a π-conjugated polymer. In particular, alkyl thiophenes have been extensively exploited for device applications such as FETs, light-emitting diodes, and sensors. A recent review article, by Mancuso and Gabriele, well describes the synthetic strategies for various thiophene derivatives [29]. Although EDOT is a well-known thiophene derivative, it has no functional groups for further modification. Therefore, EDOT has also been further modified to contain functional groups such as hydroxyl, ether, and ester groups. Figure 4 shows synthetic procedures for the synthesis of several EDOT derivatives. In addition to the possibilities for further functionalization, the derivatization of the monomer leads to different properties compared to the original monomer. Advances in polymerization techniques, such as controlled/living radical polymerization have enabled diverse polymerization reactions that yield highly functionalized polymeric materials. However, there is still a broad range of side-chain functionalities that cannot be directly incorporated into polymers by existing polymerization techniques. Such functional groups may either completely prevent controlled polymerization or may participate in side reactions that may lead to loss of control in the polymerization reaction. Consequently, most thiophene derivatives have been polymerized so far in the form of polymer films rather than nanostructures: commonly, polythiophene derivatives can be electrochemically deposited as a thin film with an appropriate dopant and then receptor molecules are covalently immobilized to the polymer. Such problems are similarly found in other kinds of f-monomers.

### 2.3. Copolymerization

To circumvent the problematic issues occurring in the polymerization of f-monomers alone, functional groups can be introduced into polymers through copolymerization. In other words, f-monomers can be introduced to the polymerization process of monomers with no functionality (n-monomers) to yield copolymers, where the degree of functionalization is controlled by adjusting the molar ratio of f-monomer to n-monomer. Similar to the homopolymerization of f-monomers, there are several issues that should be considered carefully for the successful fabrication of polymer nanostructures. As a detailed example, while the most monomers are hydrophobic, functional groups that enable subsequent chemical reactions are mostly hydrophilic. Therefore, there should be precise interfacial control between the different monomers during the fabrication of nanostructures. Additionally, as mentioned earlier, the major properties of the polymer nanoparticles are affected by the introduction of f-monomers, which may affect the sensing performance depending on the degree of functionalization. For these reasons, most researchers have prepared polymer films for sensing applications using copolymerization strategies. Foot and co-workers developed a potentiometric urea biosensor using a conductive thiophene copolymer, poly(3-hexylthiophene-*co*-3-thiopheneacetic acid) (P(3HT-3TAA)) [30]. Urease was covalently linked to the surface carboxyl group of the P(3HT-3TAA) film and the resultant urease-immobilized P(3HT-3TAA) film electrode had a detectable concentration range of about 1‒5 mM (the normal level of urea in blood serum is 1.3–3.5 mM). Travas-Sejdic used pyrrole and thiophene derivative monomers, such as carboxylic acid-bearing pyrrole and thiophene phenylenes, to immobilize receptors on the resulting polymer film [31]. Oligonucleotides were readily attached to the pyrrole-/thiophene-based termonomers using carbodiimide chemistry, and subsequent electrochemical polymerization resulted in conductive polymer thin films, which allowed selective DNA detection.

**Figure 5:**
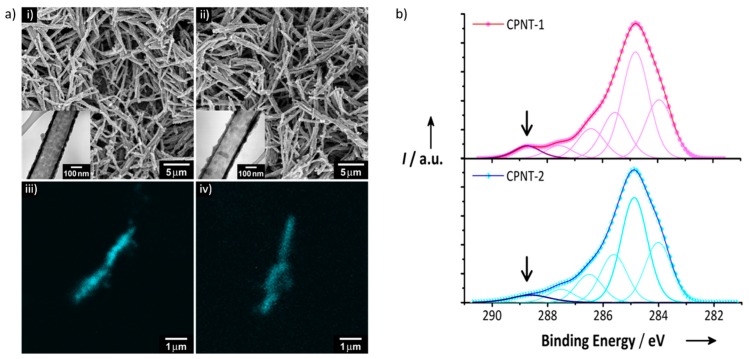
Carboxylic-acid-functionalized PPy (CPPy) nanotubes with different chemical functionalities. (**a**) (**i**,**ii**) SEM and TEM (inset) images and (**iii**,**iv**) CLSM images (λ_exc_ = 458 nm), where pyreneacetic acid was covalently conjugated to the nanotubes: (**i**,**iii**) CPNT-1 and (**ii**,**iv**) CPNT-2; (**b**) XPS C1s spectra (the arrow indicates the C1s component that originates from carboxylic acid group). Reproduced with permission from [35]. Copyright 2008, John Wiley & Sons.

Despite the difficulties in fabricating copolymer nanostructures, several works have successfully demonstrated that copolymerization can be used for the preparation of transducer polymer nanoparticles. As a notable example, Jang and Yoon developed for the first time a soft template approach to fabricate one-dimensional polymer nanostructures [32,33]. Electrically conductive polypyrrole (PPy) and PEDOT nanostructures were prepared using a reverse cylindrical micelle template comprised of sodium bis(2-ethylhexyl)sulfosuccinate (AOT) [9,10,11,34]. The cylindrical reverse micelle was robust as a template and, thus, sustainable during the chemical oxidation polymerization. As a result, the copolymerization of pyrrole or EDOT monomers with carboxyl-group-bearing counterpart monomers was successfully carried out without any template deformation. The degree of carboxyl-group functionalization in the final product was also tunable by adjusting the concentration of the carboxylated monomer. Representatively, carboxylic-acid-functionalized PPy (CPPy) nanotubes for biosensing were prepared by the AOT cylindrical micelle templating [35]. Pyrrole-*3*-carboxylic acid (P3CA) was copolymerized with pyrrole on the cylindrical micelle template, yielding well-defined nanotubes, as shown by the scanning electron microscope (SEM) and transmission electron microscope (TEM) images in Figure 5a. The introduction of the carboxyl group to the nanotubes and its availability for further functionalization were confirmed by covalently attaching pyrene molecules to the nanotube surface (see the confocal laser scanning microscopy (CLSM) images in Figure 5a). The surfaces of the nanotubes were modified with ethylenediamine, and, subsequently, pyreneacetic acid was chemically anchored to the nanotubes via amide bond formation. In addition, the chemical functionality of the nanotubes was quantitatively tuned by adjusting the P3CA-to-pyrrole molar ratio (CPNT-1, 1:15; CPNT-2, 1:30). Figure 5b shows the X-ray photoelectron spectra (XPS) of the C1s spectra of the nanotubes in CPNT-1 and CPNT-2. The intensity of the carboxylic acid group component at 288.7 eV is clearly dependent on the P3CA-to-pyrrole molar ratio.

**Figure 6:**
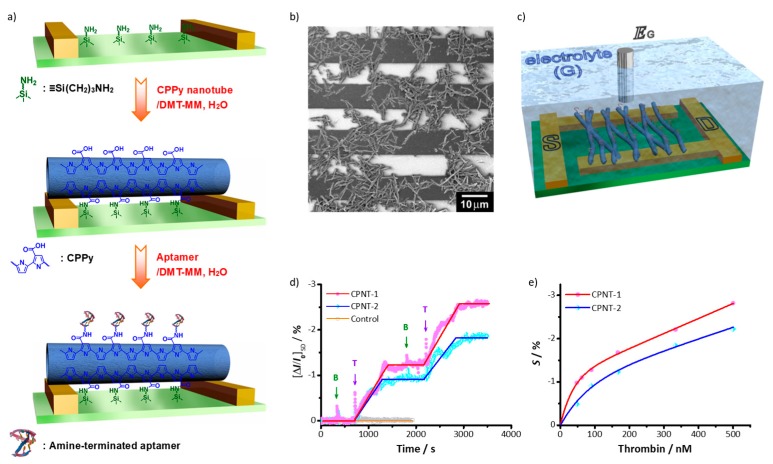
(**a**) Schematic illustration of reaction steps for the fabrication of sensor platforms based on CPPy nanotubes; (**b**) Scanning Electron Microscope (SEM) image of CPPy nanotubes that are deposited on the interdigitated microelectrode substrate; (**c**) Schematic representation of a CPPy nanotube sensor platform with a field-effect transistor (FET) configuration: the source (S), drain (D), and liquid-ion gate (G) are labelled; (**d**) Typical real-time responses of CPPy nanotube FET sensors at a constant *V*_SD_ (thrombin, T; bovine serum albumin, BSA, B), where a control experiment was performed by using CPNT-1 with no thrombin aptamers attached; (**e**) Calibration curves of CPPy nanotube FET sensors. Reproduced with permission from [35]. Copyright 2008, John Wiley & Sons.

It is important to note that the surface chemical functionality of the resultant CPPy nanotubes allows the anchoring of the nanotubes themselves to an electrode substrate, as well as the immobilization of bioreceptors on the nanotubes. Figure 6a illustrates the reaction steps for constructing a CPPy-nanotube-based sensing platform. First, the surface of the microelectrode substrate was modified with primary amino groups of an amino-silane and then the carboxyl group of the nanotubes were covalently linked with the surface amino group of the substrate through amide bond formation. Amine-terminated thrombin aptamers as the receptor were subsequently immobilized on the surface of the nanotubes via the same amide bond chemistry. Polymer nanostructures are not suitable to the conventional lithographic process for the fabrication microelectrode sensing substrates because they are highly vulnerable to the chemicals and heat used during the lithographic process. The covalent anchoring of a polymer transducer onto the microelectrode substrate was possible not just for the one-dimensional CPPy nanotubes but also for the CPPy nanoparticles [36]. Therefore, the covalent anchoring approach is an excellent alternative to construct polymer nanostructures-based microelectrode sensing platforms. Figure 6b shows the CPPy-nanotube-anchored microelectrode substrate, and Figure 6c represents a liquid-ion-gated FET sensing platform based on the CPPy nanotubes-anchored microelectrode substrate. The CPPy nanotubes anchored on the electrode substrate provided stable transistor behavior in the liquid phase, finally resulting in sensitive and selective responses toward the target species, thrombin (Figure 6d,e). Similar approaches had been used for other FET sensors, such as human olfactory receptor-based bioelectronic noses [37], enzyme-based glucose sensors [38], and heparin-based protein sensors [39]. The copolymerization strategy provides effective control of the chemical functionality of polymer nanostructures, both qualitatively and quantitatively, and, thus, may be widely applicable to various types of materials and devices.

### 2.4. Post-Polymerization Modification

A variety of functional polymers have been prepared by implementing small-molecule chemical reactions to homopolymers [40,41,42]. Diverse chemical strategies have been utilized for the post-polymerization modification, where versatile functionalities have been introduced into polymer chains by conjugating the polymer with appropriate chemical and biological species. Fundamentally, the reaction sites of f-monomers for propagation are screened by bulky functional groups and, thus, the controlled polymerization of the monomers suffers from steric hindrance. Such problems can be removed by using the post-polymerization modification strategy. Chemical strategies for the post-polymerization functionalization have been summarized in several literatures [43,44]. In particular, Klok and *co*-workers well described in detail the chemical strategies available for post-polymerization mechanism [42,44]. The chemical reactions can be categorized into several classes, as listed in Table 1, such as thiol-ene (radical thiol) addition, thiol-disulfide exchange, epoxides/anhydrides/isocyanates, ketones/aldehydes, active esters, Diels–Alder cycloaddition, and Michael addition. It is important to note that the chemical reactions requiring harmful catalysts, high temperatures, and other toxic reagents are not suitable for preparing biosensing materials.

**Figure 7:**
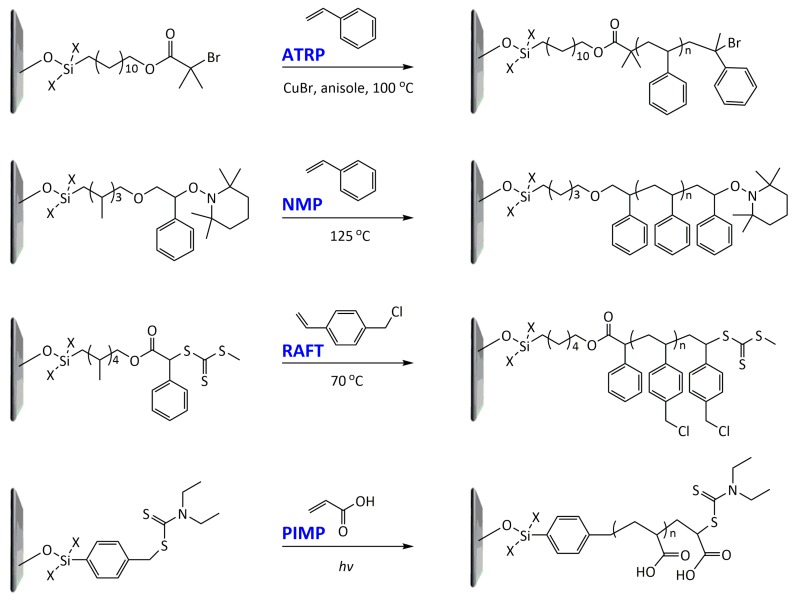
Typical examples of the surface-initiated controlled radical polymerization techniques: atom transfer radical polymerization (ATRP) of styrene, nitroxide-mediated polymerization (NMP) of styrene, reversible addition-fragmentation chain transfer polymerization (RAFT) of vinylbenzene chloride, and photoiniferter-mediated polymerization (PIMP) of acrylic acid [62,64].

Post-polymerization strategies are applicable for the functionalization of various polymer nanostructures, as well as the modification of surfaces/interfaces of interest. Representatively, polymer brushes can be introduced to the surface of a material to tailor the surface properties for biomedical and bioanalytical applications [62]. A convenient way to control the functionality of a polymer encompassing structural flexibility, chemical/biological affinity, and biocompatibility involves the modification of the polymer surface with other functional polymers via surface-initiated polymerization. The most efficient strategies for the surface-initiated polymerization involve atom transfer radical polymerization (ATRP), nitroxide-mediated polymerization (NMP), photoiniferter-mediated polymerization (PIMP), and reversible addition-fragmentation chain transfer polymerization (RAFT) [63]. Figure 7 represents typical examples of each of the surface-initiated controlled radical polymerization techniques, where the initiators were attached to the substrates through silane-based bonds [62,64]. First, ATRP has been widely used as a prominent surface-initiated polymerization method. The ATRP proceeds in a mild condition and, thus, is applicable to various biological environments. The surface-initiated ATRP has generated various polymer brushes, which are of importance in a range of bioapplications [65]. Nonspecific adsorption of biological species, such as proteins, cells, and bacteria, is mostly triggered by the chemical structure, topography, and the flexibility of the material near the surface. Importantly, nonspecific adsorption causes blood coagulation, complement activation, inflammation, biodegradation, and infection, subsequently resulting in device failure. It has been found that polymer brushes can suppress such nonspecific protein adsorption and cell adhesion [66]. In particular, poly(ethylene glycol) (PEG) and its derivatives, such as poly(oligo(ethylene glycol) methacrylate) [67,68,69,70], have been widely used to prevent undesirable bio-fouling in many applications. Other kinds of polymer grafts have been created from a range of neutral and zwitterionic polymers [71,72], including poly(2-hydroxyethyl methacrylate) [73], poly(2-methacryloyloxyethyl phosphorylcholine) [74,75,76,77], poly(sulfobetaine methacrylate) [78,79,80,81], and poly(carboxybetaine methacrylate) [82,83]. Recently, zwitterionic groups such as phosphorylcholine, sulfobetaine, and carboxybetaine have been considered to be good alternatives to conventional PEG groups owing to their enhanced antifouling effects, biocompatibility, and stability. Surface-initiated ATRP generally includes two steps. Firstly, the low-molecular-weight initiators are covalently grafted onto the surface of interest. Thiol-metal and silane-based bonds can be easily formed onto metal or metal oxide surfaces to attach the initiator. However, it is significantly more challenging to introduce the initiator on a polymeric substrate than an inorganic substrate. There are several strategies for the introduction of the initiator onto polymeric substrates. For examples, Zhao et al. covalently attached an ATRP initiator, 2-bromoisobutyryl bromide, to the hydroxyl group of hydroxymethyl 3,4-ethylenedioxythiophene (EDOT–OH) monomer via an esterification reaction [84]. Polymer brushes consisting of poly((oligo(ethylene glycol) methacrylate) and zwitterionic poly([2-(methacryloyloxy)ethyl]dimethyl-(3-sulfopropyl)ammonium hydroxide) were successfully grafted from PEDOT by ATRP. Despite the advantages of ATRP, its wide application is hindered by certain limitations. First, ATRP proceeds in the presence of a transition metal catalyst; these catalysts are generally toxic. Therefore, the catalyst must be removed prior to use in bioapplications. Secondly, acidic monomers that can react or complex with the transition metal catalyst are not amenable for ATRP. Similar to ATRP, NMP takes advantage of the reversible activation–deactivation of propagating polymer chains by a nitroxide radical. RAFT is based on reversible chain transfer with a combination of a conventional free radical initiator and a chain transfer agent. PIMP utilizes a somewhat unique class of unconventional initiators, so called iniferters that can act as initiators, chain transfer agents, and terminators for controlled polymerization. The rate of polymerization in PIMP depends on the intensity of the ultraviolet light (UV) source. The surface-initiated polymerization approach by photoirradiation allows spatial and temporal control on light exposure, providing various ways for more complicated, multidimensional, and larger-area surface/interface engineering without being limited to specific types of monomers. In addition, the use of a UV light source removes the need for transition metal catalysts that may be detrimental to the biocompatibility of the resulting polymers. These controlled/living radical polymerization methods have the advantage of simple experimental setup, mild reaction conditions, tolerance toward diverse functional groups, and compatibility with both aqueous and organic media, thus serving as powerful tools for surface and interface engineering. However, it should be noted that the elimination or minimization of biofouling alone is not sufficient for biosensing applications. In addition, appropriate receptors should be immobilized on the transducer polymer in a controlled fashion for the specific recognition of target species.

## 3. Macrostructure: Hydrogels

Hydrogels have gained interest over the past decades as promising materials in industry and daily life. They have been used as forms of hydrophilic matrices for soil conditioning, diapers, and contact lenses and have broadened the applications of nanoscience or nanoengineering with the aid of advanced analytical and processing technologies. Recently, these materials have been extensively investigated for biosensor applications such as in wearable or healthcare devices. Here, we summarize the design principles for macrostructured hydrogel biosensors. This chapter is divided into three subcategories of covalent chemistry, non-covalent chemistry, and miscellaneous approaches.

### 3.1. Covalent Chemistry for Hydrogels

#### 3.1.1. Radical Polymerization

**Figure 8:**
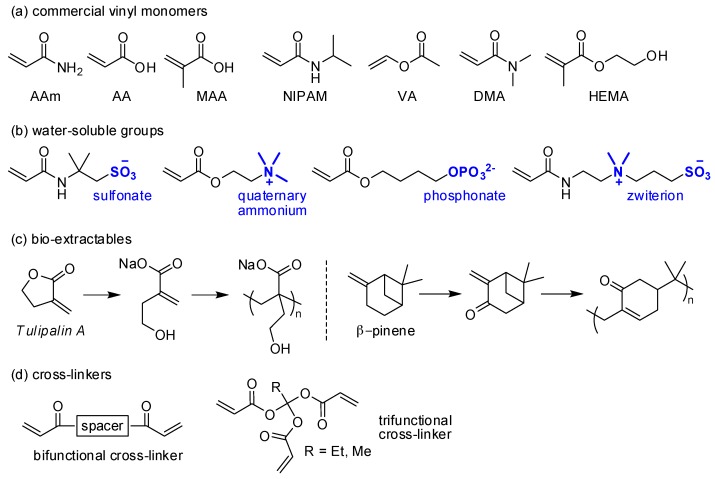
(**a**–**d**) Chemical structures of: (**a**) select vinyl monomers; (**b**) vinyl monomers that contain typical water-soluble groups; (**c**) bioextractable molecules with polymerizable groups and polymerization processes; and (**d**) bi- and trifunctional cross-linkers.

Radical polymerization is the most potent method to fabricate hydrogels among the covalent approaches. Hydrophilic monomers such as acrylamide (AAm), acrylic acid (AA), methacrylic acid (MAA), vinyl acetate (VA; the monomer for poly(vinyl alcohol), PVA), *N*,*N*-dimethylacrylamide (DMA), *N*-isopropylacrylamide (NIPAM), and 2-hydroxyethyl methacrylate (HEMA) have been widely used and polymerized by radical initiation (Figure 8a). Other monomers having water-soluble groups (e.g., carboxylate, sulfonate, quaternary ammonium, and phosphate groups, or zwitterions) are also used in hydrogel-based materials (Figure 8b). Interestingly, hydrophilic, bio-extractable monomers (e.g., Tulipalin A and β-pinene) can also be polymerized to make hydrogel polymers (Figure 8c) [85]. For cross-linking reactions, bi- or trifunctional cross-linkers are incorporated during polymerization to establish entangled network structures (Figure 8d).

**Figure 9:**
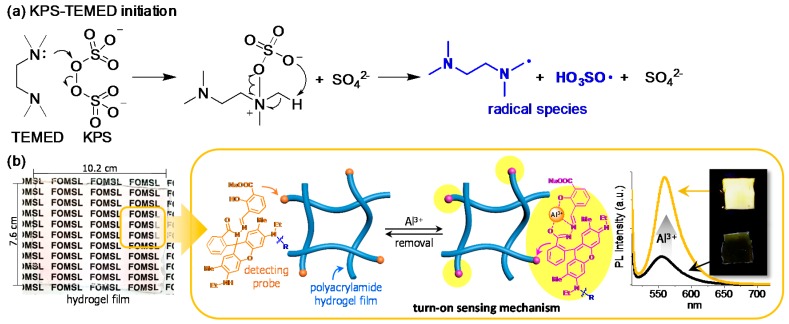
(**a**) Schematic description of the potassium persulfate-*N*,*N*,*N*,*N*-tetramethylethylenediamine (KPS–TEMED) redox system for radical initiation; (**b**) Stimuli-responsive hydrogel film having a rhodamine-based probe that induces the redox reaction for radical initiation and selectively and reversibly detects Al^3+^.

The monomeric vinyl species mentioned above are polymerized by water-soluble initiators such as persulfates (e.g., ammonium persulfate, APS; potassium persulfate, KPS) or acetophenone derivatives (e.g., IRGACURE^®^ series), which initiate polymerization after exposure to heat or light. Redox reactions also form radicals from the persulfates, initiating polymerization in the presence of a redox promoter [86,87,88,89]. A combination of KPS–TEMED (*N*,*N*,*N*,*N*-tetramethylethylenediamine) has been widely used, and other combinations have also been shown to generate radicals (Figure 9a). In particular, redox-initiated polymerization is quite useful for labeling hydrogel materials. Lee et al. demonstrated a stimuli-responsive hydrogel film labeled with a rhodamine-based probe [90]. In this system, the probe contains a secondary amine group, which plays a dual role in the film: as a redox promoter for the polymerization of AAm and as a detection probe for fluorescent sensing. The transparent hydrogel film was fabricated easily via one-step synthesis and used for the selective and reversible detection of Al^3+^, an ion that inhibits the indigestion of essential metal ions and is suspected to be a cause of Parkinson’s and Alzheimer’s disease (Figure 9b).

**Figure 10:**
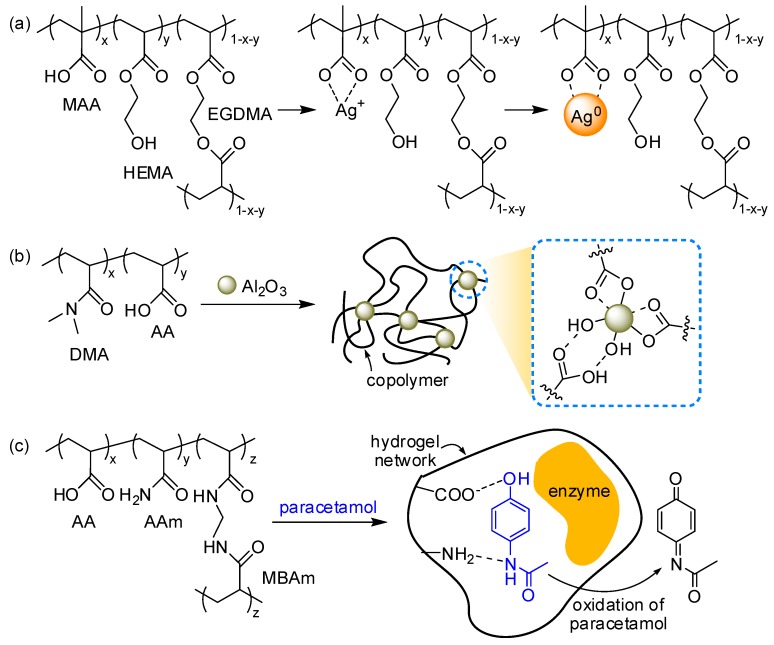
(**a**) Chemical structure of hydrogel sensor comprised of methacrylic acid (MAA), 2-hydroxyethyl methacrylate (HEMA), and ethylene dimethylacrylate (EGDMA) where silver ions are reduced to form nanoparticles; (**b**,**c**) Schematic description of (**b**) non-covalent cross-linking of poly(*N*,*N*-dimethylacrylamide-*co*-acrylic acid) by aluminum oxide nanoparticles, and (**c**) enzyme-incorporated hydrogel matrix that selectively oxidizes paracetamol and senses it electrochemically.

The copolymerization of vinyl monomers significantly alters the overall physical properties and broadens the applications of the final product [91,92,93]. AA or MAA have been extensively employed with other monomers because the carboxylic acid in AA or MAA can be used as a pH-sensitive bidentate ligand. Yetisen et al. fabricated a photonic-crystal pH sensor from a hydrogel matrix, which was prepared by copolymerizing HEMA and MAA in the presence of ethylene dimethylacrylate (EGDMA, bifunctional cross-linker). After UV polymerization, the hydrogel matrix was doped with silver ions, which were further reduced to silver nanoparticles, resulting in one-dimensional photonic crystal flakes. The free-standing flakes showed a color change while shifting the diffraction wavelength in response to pH change (Figure 10a) [81]. Similarly, Jia et al. prepared a photonic-crystal hydrogel by incorporating iron oxide nanoparticles into a hydrogel matrix of AAm, AA, and *N*,*N*-methylenebisacrylamide (MBAm). The colloidal hydrogel sensor displayed a complete color change in response to pH or ions [82]. Furthermore, exploiting the coordination ability of AA, Lu et al. developed elastic nanocomposite hydrogels without covalent cross-linking. During radical copolymerization of DMA and AA, aluminum oxide nanoparticles induced non-covalent cross-linking via secondary interactions, which gave rise to a three-dimensional colloidal array of nanocomposites that can be used to monitor the strain of materials via color change and can be applied for intraocular pressure tonometry or blood gas analysis (Figure 10b) [83]. The acid group was also used for the selective detection of paracetamol, an analgesic drug, in an electrochemical sensor. Copolymerization of AA, AAm, and MBAm afforded a hydrogel matrix containing both acidic and basic groups, which prevented the denaturation of enzyme (polyphenol oxidase, PPO) incorporated in the matrix. The oxidation of paracetamol was monitored by cyclic voltammetry (Figure 10c) [94].

**Figure 11:**
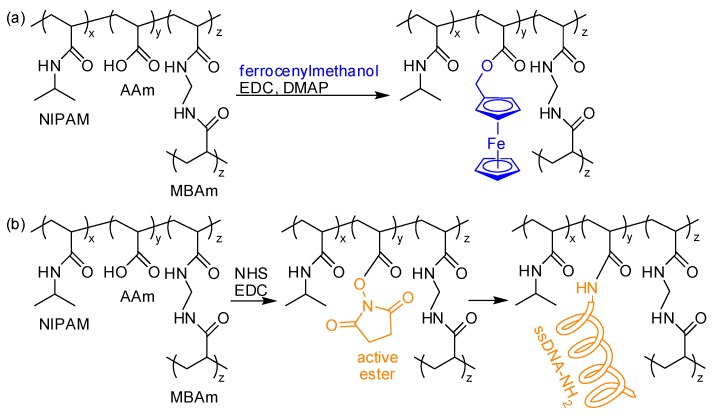
(**a**,**b**) Schemes for post-polymerization modification of hydrogel matrices via covalent bonds: (**a**) Steglich esterification using carbodiimide (EDC) and 4-dimethylaminopyridine (DMAP) to introduce ferrocenyl groups or (**b**) active ester formation using *N*-hydroxysuccinimide (NHS) to tether single-stranded DNA (ssDNA).

The acid group has been also used for post-polymerization bio-conjugation by forming ester or amide linkages. Zhang et al. prepared microgels containing AA and introduced H_2_O_2_-sensitive ferrocenyl groups via Steglich esterification using a water-soluble carbodiimide (EDC) and a 4-dimethylaminopyridine (DMAP) catalyst. Furthermore, they deposited microgels between two transparent plates to build optical devices, etalons that exhibited optical change in response to peroxides (Figure 11a) [95]. Kowalczyk et al. attached single-stranded DNA (ssDNA) onto a cross-linked hydrogel matrix containing AA repeating units. They pre-modified acid groups in the matrix with *N*-hydroxysuccinimide (NHS) to yield an active ester that sequentially reacted with amine-functionalized ssDNA in sequence under mild conditions (Figure 11b) [96].

**Figure 12:**
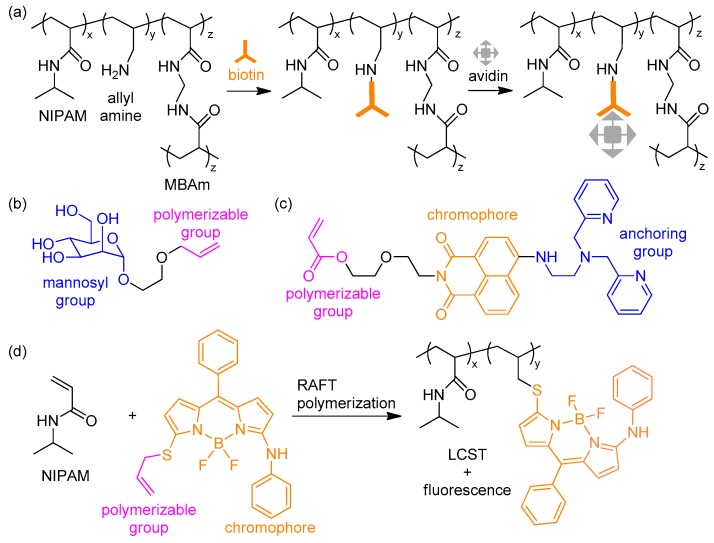
(**a**) Synthetic procedures of the biotin-containing hydrogel for label-free sensor array; (**b**,**c**) Chemical structures of (**b**) the biocompatible monomer containing a monosaccharide and (**c**) the multi-functional monomer containing a chromophore and anchoring group; (**d**) RAFT polymerization of *N*-isopropylacrylamide (NIPAM) and the fluorescent monomer. The resulting polymer shows fluorescence in response to temperature change.

Figure 12 shows other comonomers that impart hydrogel sensors with new functionalities or optimize physical properties. Miller group used allylamine to add a biotin anchoring group that selectively recognizes the protein avidin via antigen–antibody interactions. They prepared functionalized microgel particles and demonstrated a label-free sensor array using the specific interaction (Figure 12a) [97]. Song et al. also used the monomer for the encapsulation of gold nanoparticles and further developed multi-stimuli-responsive hydrogel valves that regulate the flow of water [98]. In addition, various functional monomers have been designed and polymerized. Zhang et al. synthesized allyl-substituted mannose as a comonomer where allyl groups played a role as a polymerizable group and mannosyl group as an anchor for lectin concanavalin (Con A) via lectin–carbohydrate interactions (Figure 12b). A hydrogel matrix including the monomer was polymerized and further processed to the photonic crystal that optically sensed Con A by the change in the diameter of the Debye ring [99]. Multi-functional monomers were synthesized by Hamilton et al. The monomer consists of a polymerizable fluorescent signal transducer and dipicolylamine receptor. The monomer was non-fluorescent because of photoinduced electron transfer (PET) but emitted fluorescence after the coordination of Zn^2+^ (Figure 12c) [100]. Gong et al. synthesized a functional monomer based on 4,4-difluoro-4-bora-3a,4a-diaza-*s*-indacene (BODIPY) dye containing a polymerizable allyl group. They copolymerized the monomer with NIPAM by living radical polymerization, i.e., reversible addition-fragmentation transfer (RAFT) polymerization. The resulting polymer formed a hydrogel because of the lower critical solution temperature (LCST) behavior in accordance with temperature change (Figure 12d) [101].

#### 3.1.2. Other Covalent Reactions

**Figure 13:**
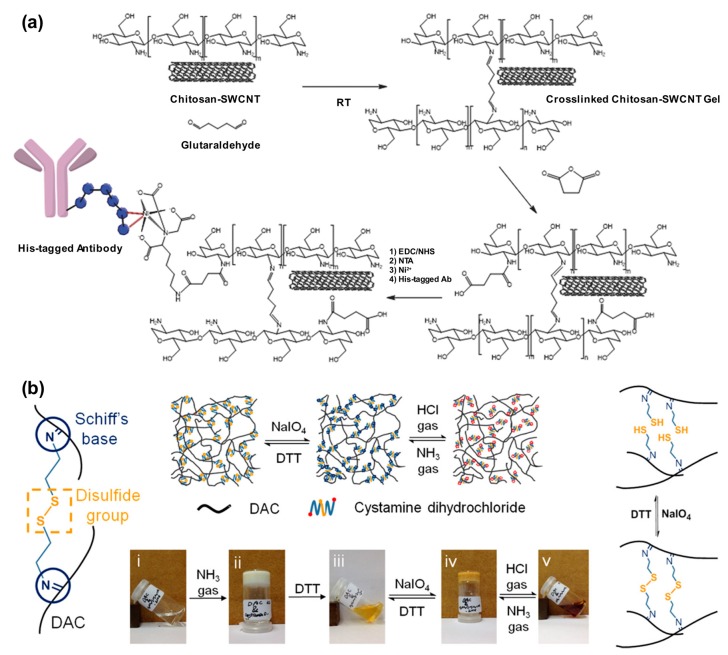
(**a**) Synthetic procedures for the single-walled carbon nanotubes (SWCNT) sensor. Chitosan is cross-linked by glutaraldehyde forming imine bonds in the presence of SWCNTs. The CNT network was further functionalized with succinic anhydride to give a carboxylic acid group that could be conjugated with His-tagged antibody using Ni(II) complexation. Reprinted with permission from [103]. Copyright (2014) John Wiley & Sons; (**b**) Depiction of redox-responsive cross-linker that provided a dual signal-responsive hydrogel. The hydrogel showed reversible transformation by redox reaction or pH because of disulfide group and imine groups respectively. Reprinted with permission from [106]. Copyright (2017) American Chemical Society.

In addition to radical polymerization, other chemical reactions that form diverse chemical linkages have been used for the preparation of hydrogels. Because radical polymerization is suitable for the construction of various polymer backbone structures, other bond-making reactions have been adopted for post-polymerization modification or cross-linking reaction via substitution or addition reactions. Among the reactions, condensation reactions are primarily used such as imine condensation. Imine bonds, specifically Schiff bases, are reversibly formed covalent bonds and can be easily controlled by an acid-catalyzed condensation reaction, eliminating a water molecule in the process. Strano group used this bond to fabricate a sensor array containing single-walled carbon nanotubes (SWCNT) that served as a point-of-care (POC) device for the detection of troponins, standard indicators of acute myocardial infarction or heart attack [102]. To achieve this, they dispersed SWCNTs in a solution of chitosan and printed the suspension using a microarray printer, which was then sequentially covered by drops of a glutaraldehyde solution that cross-linked the printed pattern (Figure 13a). Because of the robustness of the cross-linked structure, more functionalities could be introduced, such as a Ni(II) complex and histidine (His)-tagged antibody, to the SWCNT substrate, finally yielding the POC device. Besides natural polymers, other synthetic applied polymers also did not hinder the electrical performance of the nanotubes and could be used in field-effect transistors as demonstrated by Loi group [103,104,105], which would be further extended to bio-sensing fields. Another interesting example had been reported by Zhang group [106]. They exhibited multi-stimuli-responsive hydrogels based on cellulose. After periodate oxidation, the aldehyde-containing cellulose was cross-linked by a functional linker. The linker contained two amine groups for the formation of Schiff bases with the oxidized cellulose and a disulfide bond for redox-responsive behavior. Thus, the resulting hydrogel showed a reversible transformation of the shape by both pH change and redox reaction (Figure 13b).

**Figure 14:**
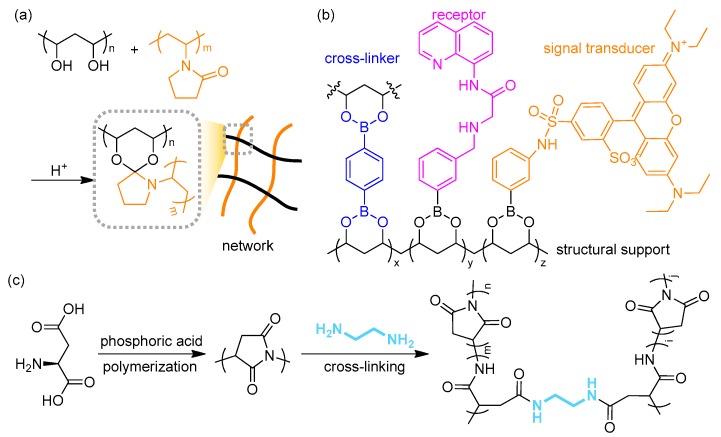
(**a**) Schematic description of the cross-linked polymer network that consist of poly(vinyl alcohol) and poly(vinylpyrrolidinone) (PVP); (**b**) Chemical structure of the transparent boronate hydrogel film containing receptor and chromophore for the selective sensing of Zn(II) ions; (**c**) Acid-catalyzed polymerization of aspartic acid and subsequent cross-linking reaction using a 1,2-diaminoethane linker by forming amide bonds.

Acetal groups are typically obtained from the condensation of an aldehyde and two equivalent alcohols. Liu et al. demonstrated that this type of reaction is efficient for the preparation of self-healing, elastic hydrogels [107]. In this reaction, the gel network consisted of PVA and poly(vinylpyrrolidinone) (PVP), which were joined together via ketal condensation (Figure 14a). Simultaneously they incorporated cellulose nanofibers with Fe^3+^, which formed the second, hard network through ionic bonding. Taking advantage of the synergistic effect from the soft and hard segments, they were able to use the double network hydrogel as a wearable strain sensor. Boronic acid also undergoes a condensation reaction to form a boronate linkage. Nishiyabu et al. used this reaction to form a multi-component, stimuli-responsive, polymer film (Figure 14b) [108]. All components such as the cross-linkers, receptors, and signal transducers containing boronic acid groups were attached to the PVA simultaneously, resulting in a transparent film that selectively detected Zn^2+^ by emitting green fluorescence. The condensation reaction of an amino acid affords biocompatible and biodegradable poly(amino acids) as a subclass of polypeptides [109]. Figure 14c shows the synthetic procedures of a colorimetric chemosensor for Cu^2+^ demonstrated by Zhang et al. [110]. The polyimide was obtained from an acid-catalyzed condensation reaction of aspartic acid, which was further cross-linked through amide bonds with ethylenediamine. They fabricated a fibrous hydrogel film based on this chemistry and could detect the presence of the target ion by the naked eye because of the colorimetric response of the material.

**Figure 15:**
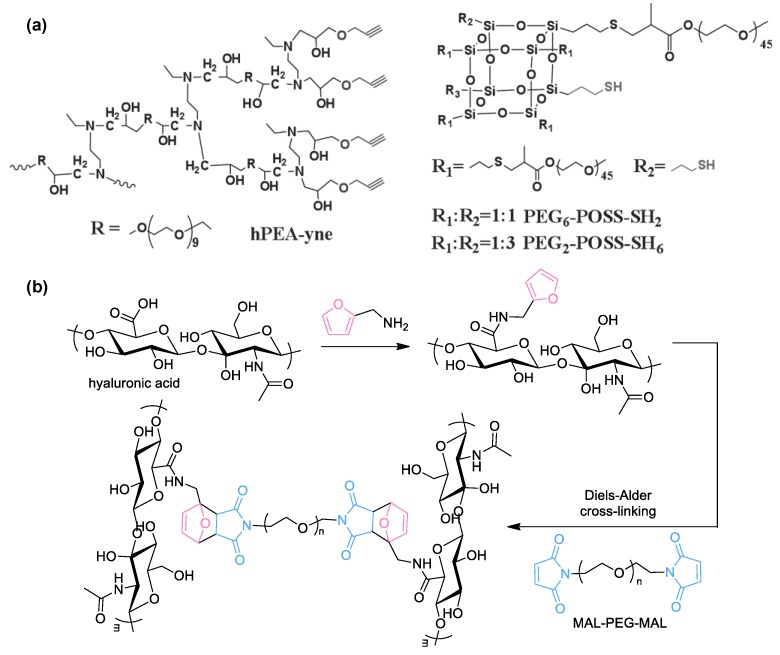
(**a**) Chemical structures of the hyperbranched polymer with terminal alkynes and the functionalized polyhedral oligomeric silsesquioxane (POSS) as macro-monomers for thiol-yne cross-linking reaction. Reprinted with permission from [111]. Copyright 2014, John Wiley & Sons; (**b**) Synthetic procedure for hyaluronic acid-poly(ethylene glycol) (HA-PEG) hydrogel via Diels–Alder click reaction. HA was functionalized with multiple furan moieties and PEG was tethered with two maleimides.

Other reactions are suitable for the preparation of hydrogels. Jiang group demonstrated the use of thiol-yne reactions for cross-linking reactions [111]. The addition reaction is photo-clickable and may further undergo a subsequent thiol-ene reaction, which is irreversible, unlike the thiol-ene reaction. They synthesized a multifunctional, hyperbranched polymer bearing peripheral acetylene groups. Through a photo-cross-linking reaction with thiol-containing polyhedral oligomeric silsesquioxane (POSS), they prepared organic–inorganic hybrid hydrogels. The gels could be patterned during UV irradiation; furthermore, residual functionalities on the patterned matrices further facilitated diverse post-cross-linking reactions, for example, thiol-based click reactions or yne-based click reactions (Figure 15a). Another notable example is the Diels–Alder reaction reported by Yu et al. (Figure 15b) [112]. Here, hyaluronic acid (HA) was pre-functionalized with furan moieties by forming amide bonds and then cross-linked with bis-maleimide-functionalized PEG (MAL–PEG-MAL) to afford biomedical HA-PEG hydrogels. Simple tuning of the ratio between furyl and maleimide moieties significantly changed the mechanical properties, and the resulting gels showed cell encapsulation viability, which could shed light on mimicking articular cartilages in terms of tissue engineering applications.

### 3.2. Non-Covalent Chemistry for Hydrogels

#### 3.2.1. Coordination Bonds

**Figure 16:**
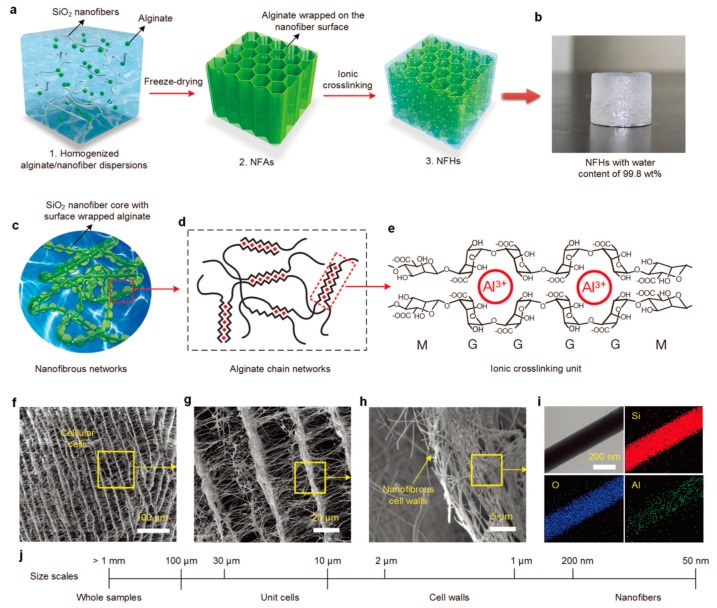
(**a**) Schematic description of the synthetic steps; (**b**) an optical photograph showing a nanofibrous network (NFH) with an ultrahigh water content of 99.8 wt %; (**c**–**e**) schematics of the three structural levels of the hydrated nanofibrous networks: alginate/SiO_2_ composite nanofibers, alginate gels, and ionic crosslinks through Al^3+^; (**f**–**h**) microscopic structure of NFHs at various magnifications demonstrating the hierarchical nanofibrous cellular architecture; (**i**) scanning tunneling electron microscopy–energy dispersive spectroscopy (STEM–EDS) images of a single nanofiber with corresponding elemental mapping images of Si, O, and Al, respectively; (**j**) the four levels of hierarchy of relevant structures in three dimensions. Reprinted with permission from [114]. Copyright (2017) John Wiley & Sons.

Non-covalent interactions facilitate facile preparation or orthogonal synthetic methodologies, giving rise to synergetic physical properties in hydrogel materials. Coordination bonds or strong secondary bonds including hydrogen bonding or hydrophobic interaction are extensively used for this purpose. Among them, metal coordination holds a key position in the fabrication of diverse polymeric hydrogels that typically include polysaccharides, for instance, alginate or κ-carrageenan. Such dynamic coordination bonding allows for easy synthesis and imparts the hydrogels with stimuli responsiveness, as well as reversibility [113]. Ding group sophisticatedly designed an alginate-based hydrogel in which a hierarchical structure was formed, containing an ultrahigh amount of water, >99 wt % [114]. In particular, SiO_2_ nanofibers played a pivotal role, while the alginate formed a three-dimensional, fibrillar framework through coordination bonds with Al^3+^, as shown in Figure 16. The resulting hydrogel was elastic and injectable, and further capable of showing pressure-sensitive conductivity.

**Figure 17:**
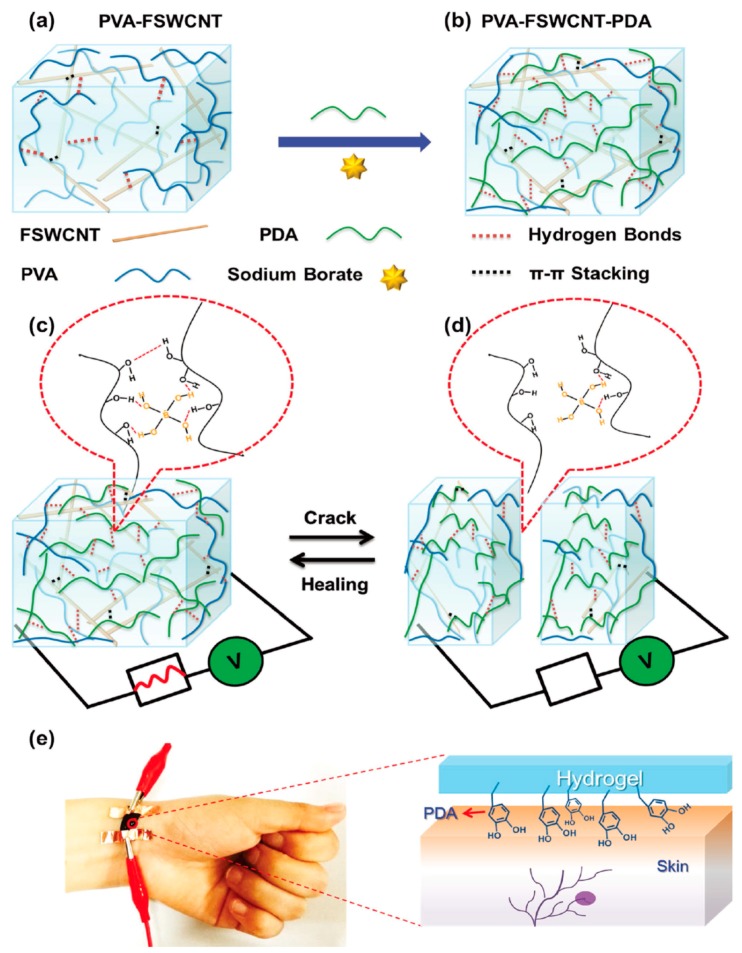
Schematic representation of the fabrication of the healable, adhesive, wearable, and soft human-motion sensors. By a delicate conformal coating of (**a**) conductive functionalized SWCNT networks through dynamic supramolecular cross-linking, (**b**) the conductive, healable, and adhesive hybrid network hydrogels are prepared. The human-motion sensors assembled from the hybrid network hydrogels could be (**c**,**d**) reversibly self-healed, and (**e**) self-adhered on the wrist during human–machine interactions and healthcare monitoring. Reprinted with permission from [115]. Copyright (2017) John Wiley & Sons.

Another interesting example is the multi-functional epidermal sensor reported by Liao et al. [115]. The sensor was fabricated based on a hydrogel comprised of multiple non-covalent bonds (e.g., coordination bonds, hydrogen bonds, and π–π stacking,), which are used as a design concept to build the entire structure and maximize the desirable properties. The reversible coordination bond between the hydroxyl group and borate is responsible for the hydrogel matrix, leading to the self-healing properties of the sensor; furthermore, the inclusion of carbon nanotubes for electrical conductivity and polydopamine for epidermal adhesion allows the strain of the hydrogel material on the skin to be determined. Therefore, the multifunctional, wearable sensor monitors the change in strain arising from human motion (Figure 17).

#### 3.2.2. Supramolecular Chemistry

**Figure 18:**
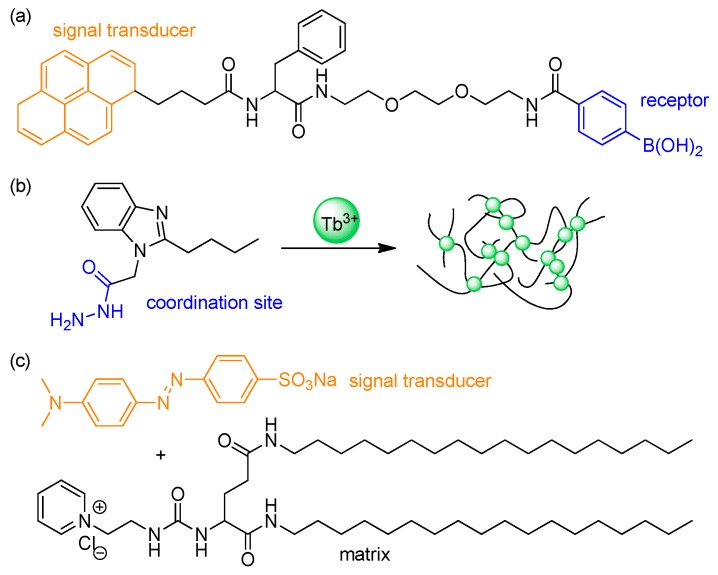
Chemical structures of: (**a**) the phenylalanine-based hydrogelator containing pyrene fluorophore and phenylboronic acid receptor; (**b**) the benzimidazole-based gelator containing a hydrazide group; and (**c**) multicomponent-gelator with a cationic gelator and an anionic methyl orange dye.

Self-assembly provides an efficient approach to form a macroscopic, well-ordered, polymeric structure that brings about other significant physical or chemical properties. Small molecules or polymers are autonomously assembled under controlled conditions that are mostly dependent on concentration or temperature, thus forming the assembled structures that contain microscopic, crystalline domains. The resulting hierarchical structures are subject to hydrogen bonding, π–π stacking, electrostatic interactions, or van der Waals interactions. As an example, Das group synthesized a small-molecule hydrogelator containing fluorescent pyrene, phenylalanine, and phenylboronic acid receptor moieties (Figure 18a). The gelator showed thermo-reversible gelation properties in water and detected glucose by changes in fluorescence [116]. Ma et al. prepared supramolecular hydrogels and investigated a reversible sol-gel transition induced by external stimuli [117]. The small-molecule gelator was synthesized from a benzimidazole derivative after functionalization with hydrazide, and further formed a hydrogel network through metal coordination with terbium(III) ions in water (Figure 18b). The resulting hydrogel showed not only sensitized luminescence but also thermo-reversibility in response to heat and pH. A multi-component hydrogel also was demonstrated by Yang et al. [118]. The system consists of a cationic organogelator and an anionic, low-molecular-weight dye. The resulting hierarchical structure can discriminate adenosine-phosphates. The addition of mono- or diphosphates such as AMP or ADP preserved the structure, although ion exchange occurred. However, in the case of the triphosphate ATP, the structure collapsed rapidly, allowing the efficient detection of triphosphate by the naked eye (Figure 18c).

**Figure 19:**
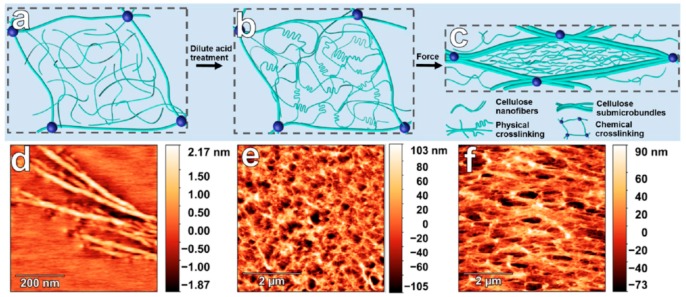
(**a**–**c**) Schematic diagram of cellulose hierarchical networks and the orientation within deformed cellulose hydrogel: (**a**) construction of loosely chemically cross-linked cellulose network as framework through reacting cellulose by adding tiny amount of cross-linker, (**b**) formation of cellulose submicrobundles and nanofibers, and (**c**) synchronized orientation of both physical and chemical networks under external force; (**d**–**f**) Representative atomic force microscopy height images. Reprinted with permission from [119]. Copyright (2017) American Chemical Society.

The crystallization of polymeric materials also induces dynamic physical cross-linking in three-dimensional networks, resulting in responsiveness to external stimuli by enhancing the mechanical properties of the hydrogel network. Zhang group incorporated cellulose nanofibers (CNF) in situ into cellulose hydrogels. The polysaccharide fibers showed concentration-dependent gelation after loose chemical cross-linking, which further progressed to induce crystalline, micro-fibrillated structures inside the gel by physical interactions. Therefore, they fabricated anisotropic, mechano-responsive hydrogels via a bottom-up approach, which switched on and off polarized light on the application of an external force (Figure 19) [119].

### 3.3. Miscellaneous Approaches

#### 3.3.1. Additives

**Figure 20:**
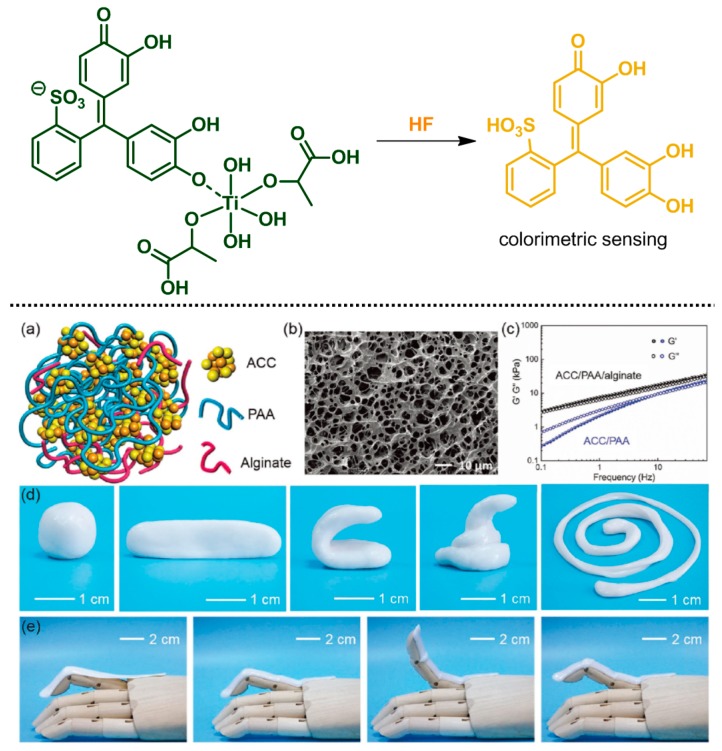
(**top**) Chemical structure of the pyrocatechol violet-based dye incorporated in the alginate hydrogel, which changed color from dark green to yellow after dissociation in response to HF gas. (**bottom**) (**a**) Schematic structure of the mineral hydrogel. (**b**) SEM image of the freeze-dried hydrogel. (**c**) Mechanical behavior of the hydrogel. (**d**) Various shapes of the hydrogel. (**e**) The hydrogel film attached to a prosthetic finger. Reproduced with permission from [124]. Copyright 2017, John Wiley & Sons.

Additives are essential, low-molecular-weight components that are incorporated into polymeric matrices to alter the performance of the entire material. The inclusion of additives enhances processability (typically having a plasticizing effect) and can render the processed materials highly functional (e.g., biodegradable, antimicrobial, fragrant, or durable under extreme conditions), as well as reducing the cost of production and satisfying the design requirements [120,121,122]. Such additives can be used in polymer hydrogels. Lee group investigated an alginate hydrogel matrix containing a pyrocatechol-violet-based dye that senses HF gas (top, Figure 20). The dye, consisting of a binding site and a signal transducer, selectively dissociated in the presence of HF in the hydrogel, which resulted in a change in color detectable by the naked eye [123]. Inorganic additives bring about synergetic effects in hydrogels. Wu group developed a self-healable ionic skin based on a hydrogel containing calcium carbonate crystals. The mineral not only induced dynamic cross-linking reaction through chelation with the carboxylate groups of poly(acrylic acid) (PAA) and alginate, but also affected ionic conductivity as a charge carrier. Thus, the artificial skin was sensitive to external pressure, for example, gentle touch or even the pressure from a small water droplet (bottom, Figure 20) [124].

#### 3.3.2. Molecular Imprinting

**Figure 21:**
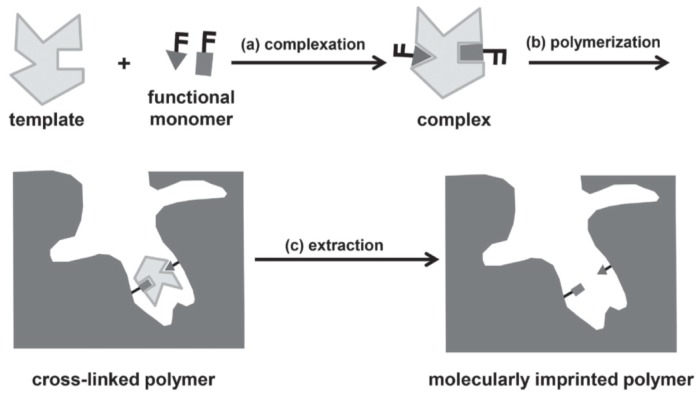
(**a**–**c**) Depiction of the preparation of a molecularly imprinted polymer including three steps: complexation (**a**); cross-linking (**b**); and the removal of template molecules (**c**). Reprinted with permission from [125]. Copyright 2014, John Wiley & Sons.

Molecular imprinting has attracted great attention over the last few decades. This approach can yield artificial receptors that feature specific recognition for target molecules, mimicking biological systems such as antibody–antigen or enzyme–substrate. The tailor-made binding sites in a molecularly imprinted polymer (MIP) are formed by: (i) complexation with templates; (ii) cross-linking reactions in the presence of the templates; and (iii) subsequent removal of the templates (Figure 21) [125]. The MIP then selectively recognizes target small molecules or even bio-macromolecules analogous to the templates. This technique can be applied to various kinds of materials including not only cross-linked powders [126] but also fibers [127] or inorganic materials [128]. In particular, a hydrogel was used as a matrix for binding target molecules [129]. EL-Sharif et al. fabricated a thin-film MIP for a quartz crystal microbalance (QCM) sensor for protein detection. They prepared the hydrogel MIP film using *N*-hydroxymethylacrylamide (NHMA) and MBAm in the presence of target proteins and deposited the film on QCM crystals by spin-casting. The film sensor displayed selective recognition through rebinding tests of protein analytes.

#### 3.3.3. Multilayered Structure

**Figure 22:**
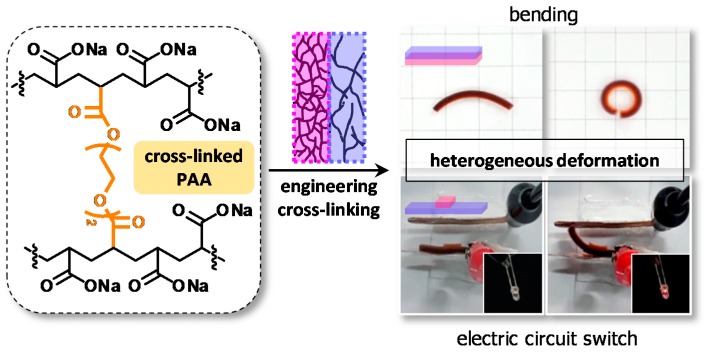
Schematic description of the fabrication of bilayered hydrogels that show heterogeneous deformation. The hydrogel bilayers were made from poly(acrylic acid) (PAA) via sequential in situ polymerization while controlling the cross-linking density. The linear bilayer curled into a round shape under aqueous conditions, and the patch-type bilayer could be used as the underwater switch.

Macroscopic responses can emerge from the structure without losing the hydrogel properties when hydrogels that display independent physical properties are integrated into a designed structure. Recently, Kim group fabricated a hydrogel actuator that consists of two hydrogel layers with different cross-linking densities. Superabsorbent PAA was used for the hydrogels. The bilayered hydrogel was fabricated via sequential in situ radical polymerization; the only difference was the cross-linking density. Hence, the bilayer showed reversible actuation under physiological conditions. When swelling, the hydrogel bilayer bent in water because of subtle differences in the cross-linking density, which was markedly dependent on the pH. Furthermore, the bilayered material was used as an underwater electronic circuit switch (Figure 22) [130]. These kinds of materials have the potential for autonomous, biomimetic applications, where materials are required to not only recognize changes in the surroundings but also provide feedback and adapt themselves to the new environment.

#### 3.3.4. Electrospinning

**Figure 23:**
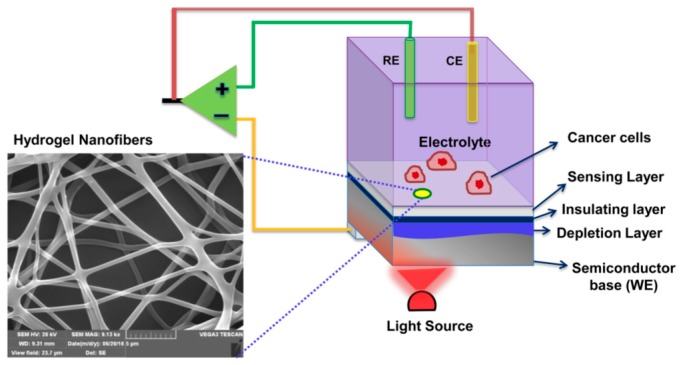
Explanatory illustration of the pH-sensitive hydrogel-nanofiber-integrated light-addressable potentiometric sensor (NF-LAPS). The pH-sensitive hydrogel was fabricated on top of a *p*-type SiO_2_ substrate as an active layer (the nanofiber layer measured by SEM). The change in photocurrent in the setup was detected while the pH was varied. Reprinted with permission from [131]. Copyright (2016) American Chemical Society.

Thundat group have demonstrated a photoelectrochemical sensor based on hydrogel nanofibers [131]. The nanofibers were deposited, via electrospinning, as an active layer and were pH-sensitive because of the presence of poly(acrylic acid). Furthermore, the device generated a photocurrent when irradiated with light (i.e., light addressable potentiometric sensor, LAPS) (Figure 23). In principle, with pH change, the fibers swell to different extents, and, thus, the device gives the corresponding current as a signal, which can be used to detect extracellular acidification.

#### 3.3.5. Hydrothermal Process

**Figure 24:**
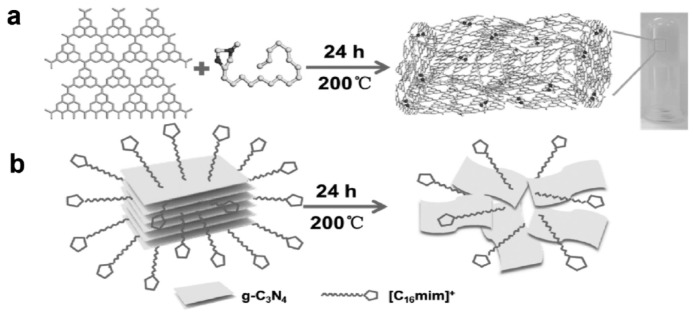
(**a**,**b**) Schematic of hydrogel formation: (**a**) the gelation occurs through IL-assisted stabilization of the exfoliated layers and (**b**) the ionic liquid drives the exfoliation of bulk carbon nitride, whereas the interactions with the nanosheets prevent restacking. Reprinted with permission from [132]. Copyright 2017, John Wiley & Sons.

The hydrothermal process has been widely studied and used to prepare 3D stacked structures. Yan et al. reported a synthetic procedure of 3D hydrogel from graphitic carbon nitrides, a graphene analog, by heating at 200 °C in the presence of amphiphilic ionic liquids (ILs); this allows the control of the morphology, size, colloidal stability, or solubility of the nanomaterial. During the process, the carbon nitrides were exfoliated by the ILs and gelled, thus forming the hydrogel network. The hydrogel was further used to prepare a film on an electrode by layer-by-layer deposition, forming a chemiresistive sensor that sensitively detected hydrogen sulfide (Figure 24) [132].

The chemical approaches discussed above including covalent chemistry, non-covalent chemistry, and other processing methods are classified by the type of reaction and functional groups, as summarized in Table 2. 

## 4. Summary and Perspectives

Compared with metals and ceramics, polymers are relatively unstable in the nanometer regime because of the nature of covalent bonds. Thus, it is not still easy to fabricate polymer nanostructures with well-defined sizes and morphologies for specific applications. In that sense, considerable attention should be paid to the functionalization of polymer nanostructures compared to the inorganic nanostructures. In addition to physical doping, many chemical routes to functionalize the polymer nanostructures for biosensing applications have been addressed in this article, which is categorized into the chemical modification of monomers, copolymerization, and post-polymerization modification. The pros and cons of each strategy should be considered to achieve the best structure and properties of polymers for biosensing applications. Nanostructures provide a high surface-to-volume ratio and small dimensions, allowing fabrication of miniaturized, highly sensitive sensors. However, it is hard to control the structure and properties of the nanostructured polymers during polymerization compared to their bulk counterparts. The use of modified monomers or comonomers before polymerization can deform the morphology of the resulting products because the interfacial tension between the monomer and polymerization medium is changed. The chemical reactivity of the modified monomers is also changed compared to non-modified, non-functionalized monomers. Therefore, much effort is required to determine the most important polymerization parameters that control the polymer properties. After polymerization, control over the morphology of the nanostructures is not required. Although the morphology is preserved without significant change, the polymer properties may be considerably altered during the post-polymerization modification reaction. The careful design of reaction procedures for post-polymerization should have no negative effects on the desired properties of the polymer. Judicious choice of experimental variables such as temperature, reaction medium, and chemicals should be made depending on the polymer type.

The macrostructures of the polymers have also been widely researched. Among them, hydrogels have attracted significant attention because of various advantages including 3D porous network, outstanding biocompatibility, good liquid permeability, chemical diversity, and facile modification, which make hydrogels ideal candidates for biosensing materials. In particular, the 3D porous matrices provide a larger contact area toward target molecules in water, and the embedding of powerful sensing tools enhances the sensing abilities of the materials. The chemical structures of hydrogel biosensors are prepared by a variety of synthetic methodologies, which have been addressed in this review in detail. Radical polymerization is a conventional yet superior method to prepare hydrogel materials together with other covalent-bond-making reactions, which play an important role under certain conditions for post-polymerization modification. Non-covalent chemistry or other miscellaneous processing approaches that result in cross-linked structures have also been introduced. Compared with molecular biosensors, hydrogel sensors tend to show a slow response time that would hinder real-time sensing because of the mass transfer of target materials inside the hydrogel matrices. Another drawback of hydrogels is their poor mechanical properties. However, these limitations can be overcome by engineering and optimizing the structure of hydrogels, which further facilitates the development of tissue engineering or wearable devices. In particular, from the perspective of biosensing, many devices that can sensitively monitor biosignals (e.g., breath, pulse, sweat, and strain) have been successfully developed based on hydrogel materials. Furthermore, new designs for hydrogels (e.g., double-network hydrogels, dynamic bonding-assisted hydrogels, and nanocomposite-reinforced hydrogels) have been extensively investigated over the last decade, and new chemical reactions such as head-to-tail depolymerization [138,139,140] or self-propagating reactions [141,142] can be further integrated into hydrogels, giving rise to an autonomous response system. In the future, hydrogels could be used as a platform for theragnosis, a combination of diagnosis and therapeutics, which is an emerging concept in biomedicine. Such hydrogel sensors would not only find early symptoms of diseases, but also visualize or release drug molecules simultaneously in response to the external changes including pH, temperature, light, and magnetic or electrical fields.

## Figures and Tables

**Table 1:** Representative post-polymerization reactions that can be used for the functionalization of polymers [42,44].

Classification	Reaction scheme	Refs.
Thiol-ene addition: The anti-Markovnikov addition of thiols to alkenes is facilitated by a radical source or by UV irradiation.	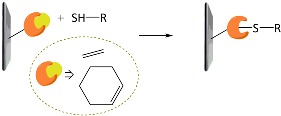	[45,46]
Thiol-disulfide exchange: This type of reaction is frequently found in biological systems. Disulfides as pyridyl disulfide are readily exchanged in high yields with thiol compounds.	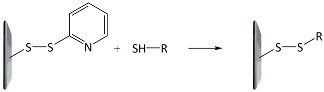	[47,48]
Epoxides, anhydrides, isocyanates: These are a class of reactive groups, that are, importantly, tolerant toward radical-based polymerization methods.	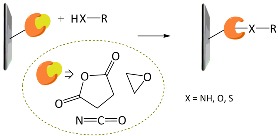	[49,50,51]
Ketones and aldehydes: These can selectively react with primary amines, alkoxyamines, and hydrazines, producing imines, oximes, and hydrozones, respectively.	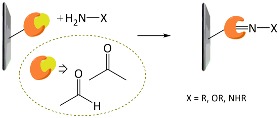	[52,53]
Active esters: The reaction of active ester groups with amines can proceeds selectively even in the presence of weaker nucleophiles, such as alcohols.	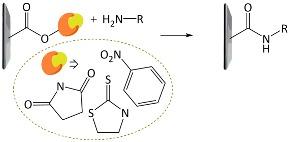	[54,55,56]
Diels–Alder cycloaddition: A diene and a substituted alkene can make cycloaddition reaction, which is reversible.	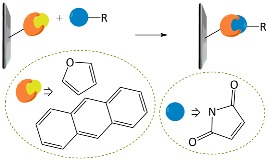	[57,58]
Michael addition: Thiols undergo Michael-type addition to activated alkenes, which proceeds rapidly in aqueous media under mild conditions.	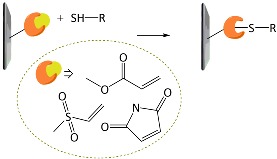	[59,60,61]

**Table 2:** Chemical approaches for the preparation of hydrogels with relevant references.

	Type of Reaction	Functional Group or Materials	Refs.
Covalent Chemistry	Radical polymerization	Vinyl monomers	[85,86,87,88,89,90,98,99]
	Radical copolymerization	Vinyl monomers	[91,92,93,94,101]
	Esterification ^1^	Carboxylic acid–alcohol	[95]
	Amidation ^1^	Carboxylic acid–amine	[96,97,106,110]
	Imine condensation ^1^	Imine–aldehydes	[102,106]
	Ketal formation ^1^	Diol–ketone	[107]
	Boron esterification	Boronic acid–diol	[98]
	Thiol-yne	Thiol–terminal alkyne	[111]
	Diels–Alder	Furan–maleimide	[112]
Non-covalent Chemistry	Metal coordination	Carboxylic acid–metal	[114,133,134,135]
		Sodium borate–diol	[115]
	Self-assembly/crystallization	Small molecule	[116,117,118]
		Polymers	[119]
Miscellaneous	Additive processing	Synthetic dye	[123]
		CaCO_3_ crystal	[124]
	Molecular imprinting	Acrylamides	[129]
	Multilayered structure	PAA	[130]
	Electrospinning	PAA	[131]
	Hydrothermal method	Graphene	[132,136,137]

^1^ Including post-polymerization modification.

## References

[1] Park S.J., Kwon O.S., Lee J.E., Jang J., Yoon H. (2014). Conducting Polymer-Based Nanohybrid Transducers: A Potential Route to High Sensitivity and Selectivity Sensors. Sensors.

[2] Yoon H., Jang J. (2009). Conducting Polymer Nanomaterials for High-Performance Sensor Applications: Issues and Challenges. Adv. Funct. Mater..

[3] Lewinski N., Colvin V., Drezek R. (2008). Cytotoxicity of Nanoparticles. Small.

[4] Zarzar L.D., Aizenberg J. (2014). Stimuli-Responsive Chemomechanical Actuation: A Hybrid Materials Approach. Acc. Chem. Res..

[5] Oliva N., Conde J., Wang K., Artzi N. (2017). Designing Hydrogels for On-Demand Therapy. Acc. Chem. Res..

[6] Annabi N., Tamayol A., Uquillas J.A., Akbari M., Bertassoni L.E., Cha C., Camci-Unal G., Dokmeci M.R., Peppas N.A., Khademhosseini A. (2014). 25th Anniversary Article: Rational Design and Applications of Hydrogels in Regenerative Medicine. Adv. Mater..

[7] Yoon H. (2013). Current Trends in Sensors Based on Conducting Polymer Nanomaterials. Nanomaterials.

[8] Park S.J., Park C.S., Yoon H. (2017). Chemo-Electrical Gas Sensors Based on Conducting Polymer Hybrids. Polymers.

[9] Yoon H., Hong J.Y., Jang J. (2007). Charge-Transport Behavior in Shape-Controlled Poly(3,4-ethylenedioxythiophene) Nanomaterials: Intrinsic and Extrinsic Factors. Small.

[10] Yoon H., Chang M., Jang J. (2007). Formation of 1D Poly(3,4-ethylenedioxythiophene) Nanomaterials in Reverse Microemulsions and Their Application to Chemical Sensors. Adv. Funct. Mater..

[11] Yoon H., Chang M., Jang J. (2006). Sensing Behaviors of Polypyrrole Nanotubes Prepared in Reverse Microemulsions: Effects of Transducer Size and Transduction Mechanism. J. Phys. Chem. B.

[12] Oh W.-K., Kim S., Yoon H., Jang J. (2010). Shape-Dependent Cytotoxicity and Proinflammatory Response of Poly(3,4-ethylenedioxythiophene) Nanomaterials. Small.

[13] Liu S., Zhang J., Dong R., Gordiichuk P., Zhang T., Zhuang X., Mai Y., Liu F., Herrmann A., Feng X. (2016). Two-Dimensional Mesoscale-Ordered Conducting Polymers. Angew. Chem. Int. Ed..

[14] Liu S., Wang F., Dong R., Zhang T., Zhang J., Zhuang X., Mai Y., Feng X. (2016). Dual-Template Synthesis of 2D Mesoporous Polypyrrole Nanosheets with Controlled Pore Size. Adv. Mater..

[15] Liu S., Gordiichuk P., Wu Z.S., Liu Z., Wei W., Wagner M., Mohamed-Noriega N., Wu D., Mai Y., Herrmann A. (2015). Patterning Two-Dimensional Free-Standing Surfaces with Mesoporous Conducting Polymers. Nat. Commun..

[16] Nguyen D.N., Yoon H. (2016). Recent Advances in Nanostructured Conducting Polymers: from Synthesis to Practical Applications. Polymers.

[17] Yoon H., Choi M., Lee K.J., Jang J. (2008). Versatile Strategies for Fabricating Polymer Nanomaterials with Controlled Size and Morphology. Macromol. Res..

[18] Molino P.J., Yue Z., Zhang B., Tibbens A., Liu X., Kapsa R.M.I., Higgins M.J., Wallace G.G. (2014). Influence of Biodopants on PEDOT Biomaterial Polymers: Using QCM-D to Characterize Polymer Interactions with Proteins and Living Cells. Adv. Mater. Interfaces.

[19] Park S.J., Song H.S., Kwon O.S., Chung J.H., Lee S.H., An J.H., Ahn S.R., Lee J.E. (2014). Human Dopamine Receptor Nanovesicles for Gate-Potential Modulators in High-Performance Field-Effect Transistor Biosensors. Sci. Rep..

[20] Jin H.J., Lee S.H., Kim T.H., Park J., Song H.S., Park T.H., Hong S. (2012). Nanovesicle-Based Bioelectronic Nose Platform Mimicking Human Olfactory Signal Transduction. Biosens. Bioelectron..

[21] Chang J.C., Brewer G.J., Wheeler B.C. (2003). A Modified Microstamping Technique Enhances Polylysine Transfer and Neuronal Cell Patterning. Biomaterials.

[22] Gilmore K.J., Kita M., Han Y., Gelmi A., Higgins M.J., Moulton S.E., Clark G.M., Kapsa R., Wallace G.G. (2009). Skeletal Muscle Cell Proliferation and Differentiation on Polypyrrole Substrates Doped with Extracellular Matrix Components. Biomaterials.

[23] Harman D.G., Gorkin R., Stevens L., Thompson B., Wagner K., Weng B., Chung J.H.Y. (2015). Poly(3,4-ethylenedioxythiophene):Dextran Sulfate (PEDOT:DS)–A Highly Processable Conductive Organic Biopolymer. Acta Biomater..

[24] Yuan G.-L., Kuramoto N. (2004). Synthesis of Helical Polyanilines Using Chondroitin Sulfate as a Molecular Template. Macromol. Chem. Phys..

[25] Collier J.H., Camp J.P., Hudson T.W., Schmidt C.E. (2000). Synthesis and Characterization of Polypyrrole–Hyaluronic Acid Composite Biomaterials for Tissue Engineering Applications. J. Biomed. Mater. Res..

[26] Stewart E.M., Liu X., Clark G.M., Kapsa R.M.I., Wallace G.G. (2012). Inhibition of Smooth Muscle Cell Adhesion and Proliferation on Heparin-Doped Polypyrrole. Acta Biomater..

[27] Yuan G.-L., Kuramoto N. (2003). Synthesis and Chiroptical Properties of Optically Active Poly(*N*-alkylanilines) Doped and Intertwined with Dextran Sulfate in Aqueous Solution. Macromolecules.

[28] Groenendaal L., Jonas F., Freitag D., Pielartzik H., Reynolds J.R. (2000). Poly(3,4-ethylenedioxythiophene) and Its Derivatives: Past, Present, and Future. Adv. Mater..

[29] Mancuso R., Gabriele B. (2014). Recent Advances in the Synthesis of Thiophene Derivatives by Cyclization of Functionalized Alkynes. Molecules.

[30] Lai C.-Y., Foot P.J.S., Brown J.W., Spearman P. (2017). A Urea Potentiometric Biosensor Based on a Thiophene Copolymer. Biosensors.

[31] Aydemir N., Chan E., Baek P., Barker D., Williams D.E., Travas-Sejdic J. (2017). New Immobilisation Method for Oligonucleotides on Electrodes Enables Highly-Sensitive, Electrochemical Label-Free Gene Sensing. Biosens. Bioelectron..

[32] Jang J., Yoon H. (2005). Formation Mechanism of Conducting Polypyrrole Nanotubes in Reverse Micelle Systems. Langmuir.

[33] Jang J., Yoon H. (2003). Facile Fabrication of Polypyrrole Nanotubes Using Reverse Microemulsion Polymerization. Chem. Commun..

[34] Jang J., Chang M., Yoon H. (2005). Chemical Sensors Based on Highly Conductive Poly(3,4-ethylenedioxythiophene) Nanorods. Adv. Mater..

[35] Yoon H., Kim J.-H., Lee N., Kim B.-G., Jang J. (2008). A Novel Sensor Platform Based on Aptamer-Conjugated Polypyrrole Nanotubes for Label-Free Electrochemical Protein Detection. ChemBioChem.

[36] Kwon O.S., Ahn S.R., Park S.J., Song H.S., Lee S.H., Lee J.S., Hong J.-Y., Lee J.S., You S.A., Yoon H. (2012). Ultrasensitive and Selective Recognition of Peptide Hormone Using Close-Packed Arrays of hPTHR-Conjugated Polymer Nanoparticles. ACS Nano.

[37] Yoon H., Lee S.H., Kwon O.S., Song H.S., Oh E.H., Park T.H., Jang J. (2009). Polypyrrole Nanotubes Conjugated with Human Olfactory Receptors: High-Performance Transducer for FET-Type Bioelectronic Nose. Angew. Chem. Int. Ed..

[38] Yoon H., Ko S., Jang J. (2008). Field-Effect-Transistor Sensor Based on Enzyme-Functionalized Polypyrrole Nanotubes for Glucose Detection. J. Phys. Chem. B.

[39] Yoon H., Jang J. (2008). A Field-Effect-Transistor Sensor Based on Polypyrrole Nanotubes Coupled with Heparin for Thrombin Detection. Mol. Cryst. Liq. Cryst..

[40] Boaen N.K., Hillmyer M.A. (2005). Post-Polymerization Functionalization of Polyolefins. Chem. Soc. Rev..

[41] Barbey R., Lavanant L., Paripovic D., Schüwer N., Sugnaux C., Tugulu S., Klok H.-A. (2009). Polymer Brushes via Surface-Initiated Controlled Radical Polymerization: Synthesis, Characterization, Properties, and Applications. Chem. Rev..

[42] Gauthier M.A., Gibson M.I., Klok H.-A. (2009). Synthesis of Functional Polymers by Post-Polymerization Modification. Angew. Chem. Int. Ed..

[43] Galvin C.J., Genzer J. (2012). Applications of Surface-Grafted Macromolecules Derived from Post-Polymerization Modification Reactions. Prog. Polym. Sci..

[44] Theato P., Klok H.-A. (2013). Functional Polymers by Post-Polymerization Modification.

[45] Kempe K., Hoogenboom R., Jaeger M., Schubert U.S. (2011). Three-Fold Metal-Free Efficient (“Click”) Reactions onto a Multifunctional Poly(2-oxazoline) Designer Scaffold. Macromolecules.

[46] Ma J., Cheng C., Wooley K.L. (2009). Cycloalkenyl-Functionalized Polymers and Block Copolymers: Syntheses via Selective RAFT Polymerizations and Demonstration of Their Versatile Reactivity. Macromolecules.

[47] Bulmus V., Woodward M., Lin L., Murthy N., Stayton P., Hoffman A. (2003). A New pH-Responsive and Glutathione-Reactive, Endosomal Membrane-Disruptive Polymeric Carrier for Intracellular Delivery of Biomolecular Drugs. J. Control. Release.

[48] Wong L., Boyer C., Jia Z., Zareie H.M., Davis T.P., Bulmus V. (2008). Synthesis of Versatile Thiol-Reactive Polymer Scaffolds via RAFT Polymerization. Biomacromolecules.

[49] Sethuraman V.A., Na K., Bae Y.H. (2006). pH-Responsive Sulfonamide/PEI System for Tumor Specific Gene Delivery: An in Vitro Study. Biomacromolecules.

[50] Barbey R., Klok H.-A. (2010). Room Temperature, Aqueous Post-Polymerization Modification of Glycidyl Methacrylate-Containing Polymer Brushes Prepared via Surface-Initiated Atom Transfer Radical Polymerization. Langmuir.

[51] Flores J.D., Shin J., Hoyle C.E., McCormick C.L. (2010). Direct RAFT Polymerization of an Unprotected Isocyanate-Containing Monomer and Subsequent Structopendant Functionalization Using “Click”-Type Reactions. Polym. Chem..

[52] Rabuka D., Parthasarathy R., Lee G.S., Chen X., Groves J.T., Bertozzi C.R. (2007). Hierarchical Assembly of Model Cell Surfaces: Synthesis of Mucin Mimetic Polymers and Their Display on Supported Bilayers. J. Am. Chem. Soc..

[53] Xiao Z.-P., Cai Z.-H., Liang H., Lu J. (2010). Amphiphilic Block Copolymers with Aldehyde and Ferrocene-Functionalized Hydrophobic Block and Their Redox-Responsive Micelles. J. Mater. Chem..

[54] Desai A., Atkinson N., Rivera F., Devonport W., Rees I., Branz S.E., Hawker C.J. (2000). Hybrid Dendritic–Linear Graft Copolymers: Steric Considerations in “Coupling to” Approach. J. Polym. Sci. A.

[55] Šubr V., Ulbrich K. (2006). Synthesis and Properties of New *N*-(2-hydroxypropyl)methacrylamide Copolymers Containing Thiazolidine-2-thione Reactive Groups. React. Funct. Polym..

[56] Hwang J., Li R.C., Maynard H.D. (2007). Well-Defined Polymers with Activated Ester and Protected Aldehyde Side Chains for Bio-functionalization. J. Control. Release.

[57] Jones J.R., Liotta C.L., Collard D.M., Schiraldi D.A. (1999). Cross-Linking and Modification of Poly(ethylene terephthalate-co-2,6-anthracenedicarboxylate) by Diels−Alder Reactions with Maleimides. Macromolecules.

[58] Canadell J., Fischer H., De With G., van Benthem R.A.T.M. (2010). Stereoisomeric Effects in Thermo-Remendable Polymer Networks Based on Diels–Alder Crosslink Reactions. J. Polym. Sci. A.

[59] Ohsawa S., Morino K., Sudo A., Endo T. (2011). Synthesis of a Reactive Polyester Bearing α,β-Unsaturated Ketone Groups by Anionic Alternating Copolymerization of Epoxide and Bicyclic Bis(γ-butyrolactone) Bearing Isopropenyl Group. Macromolecules.

[60] Yang S.K., Weck M. (2009). Covalent and Orthogonal Multi-functionalization of Terpolymers. Soft Matter.

[61] Wang R., Chen W., Meng F., Cheng R., Deng C., Feijen J., Zhong Z. (2011). Unprecedented Access to Functional Biodegradable Polymers and Coatings. Macromolecules.

[62] Zoppe J.O., Ataman N.C., Mocny P., Wang J., Moraes J., Klok H.-A. (2017). Surface-Initiated Controlled Radical Polymerization: State-of-the-Art, Opportunities, and Challenges in Surface and Interface Engineering with Polymer Brushes. Chem. Rev..

[63] Zhou X., Liu X., Xie Z., Zheng Z. (2011). 3D-patterned Polymer Brush Surfaces. Nanoscale.

[64] Wang Y., Hu S., Brittain W.J. (2006). Polymer Brush Grafted from an Allylsilane-Functionalized Surface. Macromolecules.

[65] Huang C.-F. (2016). Surface-Initiated Atom Transfer Radical Polymerization for Applications in Sensors, Non-Biofouling Surfaces and Adsorbents. Polym. J..

[66] Brown A.A., Azzaroni O., Fidalgo L.M., Huck W.T.S. (2009). Polymer Brush Resist for Responsive Wettability. Soft Matter.

[67] Ma H., Hyun J., Stiller P., Chilkoti A. (2004). “Non-Fouling” Oligo(ethylene glycol)—Functionalized Polymer Brushes Synthesized by Surface-Initiated Atom Transfer Radical Polymerization. Adv. Mater..

[68] Hucknall A., Rangarajan S., Chilkoti A. (2009). In Pursuit of Zero: Polymer Brushes that Resist the Adsorption of Proteins. Adv. Mater..

[69] Ma H., Li D., Sheng X., Zhao B., Chilkoti A. (2006). Protein-Resistant Polymer Coatings on Silicon Oxide by Surface-Initiated Atom Transfer Radical Polymerization. Langmuir.

[70] Tugulu S., Klok H. (2008). Stability and Nonfouling Properties of Poly(poly(ethylene glycol) methacrylate) Brushes under Cell Culture Conditions. Biomacromolecules.

[71] Matyjaszewski K., Tsarevsky N.V. (2007). “Green” Atom Transfer Radical Polymerization: From Process Design to Preparation of Well-Defined Environmentally Friendly Polymeric Materials. Chem. Rev..

[72] Cao Z., Gordiichuk P.I., Loos K., Sudhölter E.J.R., de Smet L.C.P.M. (2016). The Effect of Guanidinium Functionalization on the Structural Properties and Anion Affinity of Polyelectrolyte Multilayers. Soft Matter.

[73] Bhat R.R., Chaney B.N., Rowley J., Liebmann-Vinson A., Genzer J. (2005). Tailoring Cell Adhesion Using Surface-Grafted Polymer Gradient Assemblies. Adv. Mater..

[74] Monge S., Canniccioni B., Graillot A., Bobin J. (2011). Phosphorus-Containing Polymers: A Great Opportunity for the Biomedical Field. Biomacromolecules.

[75] Kobayashi M., Terayama Y., Yamaguchi H., Terada M., Murakami D., Ishihara K., Takahara A. (2012). Wettability and Antifouling Behavior on the Surfaces of Superhydrophilic Polymer Brushes. Langmuir.

[76] Nishizawa K., Konno T., Takai M., Ishihara K. (2008). Bioconjugated Phospholipid Polymer Biointerface for Enzyme-Linked Immunosorbent Assay. Biomacromolecules.

[77] Xu Y., Takai M., Ishihara K. (2009). Suppression of Protein Adsorption on a Charged Phospholipid Polymer Interface. Biomacromolecules.

[78] Zhang Z., Chen S., Chang Y., Jiang S. (2006). Surface Grafted Sulfobetaine Polymers via Atom Transfer Radical Polymerization as Superlow Fouling Coatings. J. Phys. Chem. B.

[79] Chang Y., Chang W., Shih Y., Wei T., Hsiue G. (2011). Zwitterionic Sulfobetaine-Grafted Poly(vinylidene fluoride) Membrane with Highly Effective Blood Compatibility via Atmospheric Plasma-Induced Surface Copolymerization. ACS Appl. Mater. Interfaces.

[80] Chang Y., Liao S., Higuchi A., Ruaan R., Chu C., Chen W. (2008). A Highly Stable Nonbiofouling Surface with Well-Packed Grafted Zwitterionic Polysulfobetaine for Plasma Protein Repulsion. Langmuir.

[81] Kuo W., Wang M., Chien H., Wei T., Lee C., Tsai W. (2011). Surface Modification with Poly(sulfobetaine methacrylate-co-acrylic acid) To Reduce Fibrinogen Adsorption, Platelet Adhesion, and Plasma Coagulation. Biomacromolecules.

[82] Zhang Z., Chen S., Jiang S. (2006). Dual-Functional Biomimetic Materials: Nonfouling Poly(carboxybetaine) with Active Functional Groups for Protein Immobilization. Biomacromolecules.

[83] Yang W., Xue H., Li W., Zhang J., Jiang S. (2009). Pursuing “Zero” Protein Adsorption of Poly(carboxybetaine) from Undiluted Blood Serum and Plasma. Langmuir.

[84] Zhao H., Zhu B., Luo S.-C., Lin H.-A., Nakao A., Yamashita Y., Yu H.-H. (2013). Controlled Protein Absorption and Cell Adhesion on Polymer-Brush-Grafted Poly(3,4-ethylenedioxythiophene) Films. ACS Appl. Mater. Interfaces.

[85] Nakajima H., Dijkstra P., Loos K. (2017). The Recent Developments in Biobased Polymers toward General and Engineering Applications: Polymers that Are Upgraded from Biodegradable Polymers, Analogous to Petroleum-Derived Polymers, and Newly Developed. Polymers.

[86] Li L., Wang Y., Pan L., Shi Y., Cheng W., Shi Y., Yu G. (2015). A Nanostructured Conductive Hydrogels-Based Biosensor Platform for Human Metabolite Detection. Nano Lett..

[87] Jia X., Wang J., Wang K., Zhu J. (2015). Highly Sensitive Mechanochromic Photonic Hydrogels with Fast Reversibility and Mechanical Stability. Langmuir.

[88] Xiao M., Li Y., Zhao J., Wang Z., Gao M., Gianneschi N.C., Dhinojwala A., Shawkey M.D. (2016). Stimuli-Responsive Structurally Colored Films from Bioinspired Synthetic Melanin Nanoparticles. Chem. Mater..

[89] Liu Y., Shen T., Hu L., Gong H., Chen C., Chen X., Cai C. (2017). Development of a Thermosensitive Molecularly Imprinted Polymer Resonance Light Scattering Sensor for Rapid and Highly Selective Detection of Hepatitis A Virus in Vitro. Sens. Actuators B.

[90] Lee K.M., Oh Y., Chang J.Y., Kim H. (2018). Facile Fluorescent Labeling of a Polyacrylamide-Based Hydrogel Film via Radical Initiation Enables Selective and Reversible Detection of Al^3+^. J. Mater. Chem. B.

[91] Yetisen A.K., Butt H., Yun S.-H. (2016). Photonic Crystal Flakes. ACS Sens..

[92] Jia X., Wang K., Wang J., Hu Y., Shen L., Zhu J. (2016). Full-Color Photonic Hydrogels for pH and Ionic Strength Sensing. Eur. Polym. J..

[93] Lu W., Li H., Huo B., Meng Z., Xue M., Qiu L., Ma S., Yan Z., Piao C., Ma X. (2016). Full-Color Mechanical Sensor Based on Elastic Nanocomposite Hydrogels Encapsulated Three-Dimensional Colloidal Arrays. Sens. Actuators B.

[94] Aliabadi A., Rounaghi G.H., Zavar M.H.A. (2017). A New Droplet-Based Polymeric Banana Electrochemical Biosensor for Analysis of One Microliter Solution of Paracetamol. Sens. Actuators B.

[95] Zhang Q.M., Berg D., Duan J., Mugo S.M., Serpe M.J. (2016). Optical Devices Constructed from Ferrocene-Modified Microgels for H_2_O_2_ Sensing. ACS Appl. Mater. Interfaces.

[96] Kowalczyk A., Wagner B., Karbarz M., Nowicka A.M. (2015). A Dual DNA Biosensor Based on Two Redox Couples with a Hydrogel Sensing Platform Functionalized with Carboxyl Groups and Gold Nanoparticles. Sens. Actuators B.

[97] Lifson M.A., Carter J.A., Miller B.L. (2015). Functionalized Polymer Microgel Particles Enable Customizable Production of Label-Free Sensor Arrays. Anal. Chem..

[98] Song J.E., Cho E.C. (2016). Dual-responsive and Multifunctional Plasmonic Hydrogel Valves and Biomimetic Architectures Formed with Hydrogel and Gold Nanocolloids. Sci. Rep..

[99] Zhang J.-T., Cai Z., Kwak D.H., Liu X., Asher S.A. (2014). Two-Dimensional Photonic Crystal Sensors for Visual Detection of Lectin Concanavalin A. Anal. Chem..

[100] Hamilton G.R.C., Sheng Y., Callan B., Donnelly R.F., Callan J.F. (2015). A Hydrogel Based Zinc(II) Sensor for Use in Fluorescent Multi-Well Plate Analysis. New J. Chem..

[101] Gong D., Cao T., Han S.-C., Zhu X., Iqbal A., Liu W., Qin W., Guo H. (2017). Fluorescence Enhancement Thermoresponsive Polymer Luminescent Sensors Based on BODIPY for Intracellular Temperature. Sens. Actuators B.

[102] Zhang J., Kruss S., Hilmer A.J., Shimizu S., Schmois Z., De La Cruz F., Barone P.W., Reuel N.F., Heller D.A., Strano M.S. (2014). A Rapid, Direct, Quantitative, and Label-Free Detector of Cardiac Biomarker Troponin T Using Near-Infrared Fluorescent Single-Walled Carbon Nanotube Sensors. Adv. Healthcare Mater..

[103] Gomulya W., Costanzo G.D., de Carvalho E.J., Bisri S.Z., Derenskyi V., Fritsch M., Fröhlich N., Allard S., Gordiichuk P., Herrmann A. (2013). Semiconducting Single-Walled Carbon Nanotubes on Demand by Polymer Wrapping. Adv. Mater..

[104] Bisri S.Z., Gao J., Derenskyi V., Gomulya W., Iezhokin I., Gordiichuk P., Herrmann A., Loi M.A. (2012). High Performance Ambipolar Field-Effect Transistor of Random Network Carbon Nanotubes. Adv. Mater..

[105] Derenskyi V., Gomulya W., Rios J.M., Fritsch M., Fröhlich N., Jung S., Allard S., Bisri S.Z., Gordiichuk P., Herrmann A. (2014). Carbon Nanotube Network Ambipolar Field-Effect Transistors with 108 On/Off Ratio. Adv. Mater..

[106] Liu P., Mai C., Zhang K. (2017). Formation of Uniform Multi-Stimuli-Responsive and Multiblock Hydrogels from Dialdehyde Cellulose. ACS Sustain. Chem. Eng..

[107] Liu Y.-J., Cao W.-T., Ma M.-G., Wan P. (2017). Ultrasensitive Wearable Soft Strain Sensors of Conductive, Selfhealing, and Elastic Hydrogels with Synergistic “Soft and Hard” Hybrid Networks. ACS Appl. Mater. Interfaces.

[108] Nishiyabu R., Ushikubo S., Kamiya Y., Kubo Y. (2014). A Boronate Hydrogel Film Containing Organized Two-Component Dyes as a Multicolor Fluorescent Sensor for Heavy Metal Ions in Water. J. Mater. Chem. A.

[109] Thombre S.M., Sarwade B.D. (2005). Synthesis and Biodegradability of Polyaspartic Acid: A Critical Review. J. Macromol. Sci. A.

[110] Zhang C., Wan L.Y., Wu S., Wu D., Qin X., Ko F. (2015). A Reversible Colorimetric Chemosensor for Naked-Eye Detection of Copper Ions Using Poly(aspartic acid) Nanofibrous Hydrogel. Dyes Pigments.

[111] Xu Y., Xu H., Jiang X., Yin J. (2014). Versatile Functionalization of the Micropatterned Hydrogel of Hyperbranched Poly(ether amine) Based on “Thiol-yne” Chemistry. Adv. Funct. Mater..

[112] Yu F., Cao X., Li Y., Zeng L., Zhu J., Wang G., Chen X. (2014). Diels–Alder Crosslinked HA/PEG Hydrogels with High Elasticity and Fatigue Resistance for Cell Encapsulation and Articular Cartilage Tissue Repair. Polym. Chem..

[113] Dragan E.S. (2014). Design and Applications of Interpenetrating Polymer Network Hydrogels. A Review. Chem. Eng. J..

[114] Si Y., Wang L., Wang X., Tang N., Yu J., Ding B. (2017). Ultrahigh-Water-Content, Superelastic, and Shape-Memory Nanofiber-Assembled Hydrogels Exhibiting Pressure-Responsive Conductivity. Adv. Mater..

[115] Liao M., Wan P., Wen J., Gong M., Wu X., Wang Y., Shi R., Zhang L. (2017). Wearable, Healable, and Adhesive Epidermal Sensors Assembled from Mussel-Inspired Conductive Hybrid Hydrogel Framework. Adv. Funct. Mater..

[116] Mandal D., Mandal S.K., Ghosh M., Das P.K. (2015). Phenylboronic Acid Appended Pyrene-Based Low-Molecular-Weight Injectable Hydrogel: Glucose-Stimulated Insulin Release. Chem. Eur. J..

[117] Ma X., Yu D., Tang N., Wu J. (2014). Tb^3+^-Containing Supramolecular Hydrogels: Luminescence Properties and Reversible Sol–Gel Transitions Induced by External Stimuli. Dalton Trans..

[118] Yang D., Liu C., Zhang L., Liu M. (2014). Visualized Discrimination of ATP from ADP and AMP through Collapse of Supramolecular Gels. Chem. Commun..

[119] Ye D., Cheng Q., Zhang Q., Wang Y., Chang C., Li L., Peng H., Zhang L. (2017). Deformation Drives Alignment of Nanofibers in Framework for Inducing Anisotropic Cellulose Hydrogels with High Toughness. ACS Appl. Mater. Interfaces.

[120] Hahladakis J.N., Velis C.A., Weber R., Iacovidou E., Purnell P. (2018). An Overview of Chemical Additives Present in Plastics: Migration, Release, Fate and Environmental Impact during Their Use, Disposal and Recycling. J. Hazard. Mater..

[121] Kim H., Chang J.Y. (2014). Reversible Thermochromic Polymer Film Embedded with Fluorescent Organogel Nanofibers. Langmuir.

[122] Kim H., Ryu J.H., Kim H.K., Chang J.Y. (2017). A Versatile Platform for Lanthanide(III)-Containing Organogelators: Fabrication of the Er(III)-Incorporated Polymer Nanocomposite from an Organogel Template. New J. Chem..

[123] Lee C., Ko Y.-J., Lee S.-Y. (2016). A Pyrocatechol Violet-Titanium Alkoxide Complex for HF Sensing: Study on the Complex Structure and Application. Dyes Pigments.

[124] Lei Z., Wang Q., Sun S., Zhu W., Wu P. (2017). A Bioinspired Mineral Hydrogel as a Self-Healable, Mechanically Adaptable Ionic Skin for Highly Sensitive Pressure Sensing. Adv. Mater..

[125] Kim H., Kim Y., Chang J.Y. (2014). Polymers for Luminescent Sensing Applications. Macromol. Chem. Phys..

[126] Kim H., Cha M.C., Park H.W., Chang J.Y. (2013). Preparation of a Yb(III)-Incorporated Porous Polymer by Post-Coordination: Enhancement of Gas Adsorption and Catalytic Activity. J. Polym. Sci. A.

[127] Kim Y., Chang J.Y. (2016). Fabrication of a Fluorescent Sensor by Organogelation: CdSe/ZnS Quantum Dots Embedded Molecularly Imprinted Organogel Nanofibers. Sens. Actuators B.

[128] Kim Y., Lee K.M., Chang J.Y. (2017). Highly Luminescent Tetra(biphenyl-4-yl)ethene-grafted Molecularly Imprinted Mesoporous Silica Nanoparticles for Fluorescent Sensing of Diethylstilbestrol. Sens. Actuators B.

[129] EL-Sharif H.F., Aizawa H., Reddy S.M. (2015). Spectroscopic and Quartz Crystal Microbalance (QCM) Characterisation of Protein-Based MIPs. Sens. Actuators B.

[130] Lee K.M., Kim H.J., Jung D., Oh Y., Lee H., Han C., Chang J.Y., Kim H. (2018). Rapid Accessible Fabrication and Engineering of Bilayered Hydrogels: Revisiting the Cross-Linking Effect on Superabsorbent Poly(acrylic acid). ACS Omega.

[131] Shaibani P.M., Etayash H., Naicker S., Kaur K., Thundat T. (2017). Metabolic Study of Cancer Cells Using a pH Sensitive Hydrogel Nanofiber Light Addressable Potentiometric Sensor. ACS Sens..

[132] Yan J., Rodrigues M.-T.F., Song Z., Li H., Xu H., Liu H., Wu J., Xu Y., Song Y., Liu Y. (2017). Reversible Formation of g-C_3_N_4_ 3D Hydrogels through Ionic Liquid Activation: Gelation Behavior and Room-Temperature Gas-Sensing Properties. Adv. Funct. Mater..

[133] Seo S., Lee J., Kwon M.S., Seo D., Kim J. (2015). Stimuli-Responsive Matrix-Assisted Colorimetric Water Indicator of Polydiacetylene Nanofibers. ACS Appl. Mater. Interfaces.

[134] Gogoi N., Barooah M., Majumdar G., Chowdhury D. (2015). Carbon Dots Rooted Agarose Hydrogel Hybrid Platform for Optical Detection and Separation of Heavy Metal Ions. ACS Appl. Mater. Interfaces.

[135] Qing Z., Mao Z., Qing T., He X., Zou Z., He D., Shi H., Huang J., Liu J., Wang K. (2014). Visual and Portable Strategy for Copper(II) Detection Based on a Striplike Poly(Thymine)-Caged and Microwell-Printed Hydrogel. Anal. Chem..

[136] Hoa L.T., Chung J.S., Hur S.H. (2016). A Highly Sensitive Enzyme-Free Glucose Sensor Based on Co_3_O_4_ Nanoflowers and 3D Graphene Oxide Hydrogel Fabricated via Hydrothermal Synthesis. Sens. Actuators B.

[137] Yuan M., Liu A., Zhao M., Dong W., Zhao T., Wang J., Tang W. (2014). Bimetallic PdCu Nanoparticle Decorated Three-dimensional Graphene Hydrogel for Non-Enzymatic Amperometric Glucose Sensor. Sens. Actuators B.

[138] Kim H., Mohapatra H., Phillips S.T. (2015). Rapid, On-Command Debonding of Stimuli-Responsive Cross-Linked Adhesives by Continuous, Sequential Quinone Methide Elimination Reactions. Angew. Chem. Int. Ed..

[139] Baker M.S., Kim H., Olah M.G., Lewis G.G., Phillips S.T. (2015). Depolymerizable Poly(benzyl ether)-Based Materials for Selective Room Temperature Recycling. Green Chem..

[140] Yeung K., Kim H., Mohapatra H., Phillips S.T. (2015). Surface-Accessible Detection Units in Self-Immolative Polymers Enable Translation of Selective Molecular Detection Events into Amplified Responses in Macroscopic, Solid-State Plastics. J. Am. Chem. Soc..

[141] Mohapatra H., Kim H., Phillips S.T. (2015). Stimuli-Responsive Polymer Film that Autonomously Translates a Molecular Detection Event into a Macroscopic Change in Its Optical Properties via a Continuous, Thiol-Mediated Self-Propagating Reaction. J. Am. Chem. Soc..

[142] Kim H., Baker M.S., Phillips S.T. (2015). Polymeric Materials that Convert Local Fleeting Signals into Global Macroscopic Responses. Chem. Sci..

